# Nanorough Is Not Slippery Enough: Implications on Shedding and Heat Transfer

**DOI:** 10.1021/acsami.3c14232

**Published:** 2024-01-02

**Authors:** Daniel Orejon, Yota Maeda, Peng Zhang, Fengyong Lv, Yasuyuki Takata

**Affiliations:** † Institute for Multiscale Thermofluids, School of Engineering, University of Edinburgh, Scotland EH9 3BF, United Kingdom; ‡ International Institute for Carbon-Neutral Energy Research (WPI-I2CNER), Kyushu University, 744 Motooka, Nishi-ku, Fukuoka 819-0395, Japan; § Department of Mechanical Engineering, Thermofluid Physics Laboratory, Kyushu University, 744 Motooka, Nishi-ku, Fukuoka 819-0395, Japan; ∥ Institute of Refrigeration and Cryogenics, Shanghai Jiao Tong University, Shanghai 200240, China; ⊥ School of Urban Construction and Safety Engineering, Shanghai Institute of Technology, Shanghai 201418, China

**Keywords:** low-adhesion, lubricant-infused surface LIS, condensation, heat transfer, thermal resistance

## Abstract

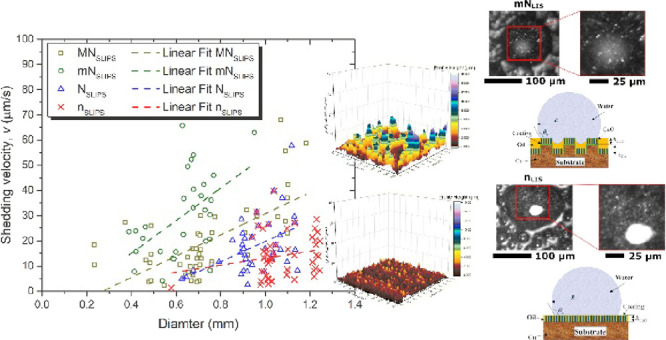

Lowering droplet–surface interactions via the implementation of lubricant-infused surfaces (LISs) has received important attention in the past years. LISs offer enhanced droplet mobility with low sliding angles and the recently reported slippery Wenzel state, among others, empowered by the presence of the lubricant infused in between the structures, which eventually minimizes the direct interactions between liquid droplets and LISs. Current strategies to increase heat transfer during condensation phase-change relay on minimizing the thickness of the coating as well as enhancing condensate shedding. While further surface structuring may impose an additional heat transfer resistance, the presence of micro-structures eventually reduces the effective condensate–surface intimate interactions with the consequently decreased adhesion and enhanced shedding performance, which is investigated in this work. This is demonstrated by macroscopic and optical microscopy condensation experimental observations paying special attention at the liquid–lubricant and liquid–solid binary interactions at the droplet–LIS interface, which is further supported by a revisited force balance at the droplet triple contact line. Moreover, the occurrence of a condensation–coalescence–shedding regime is quantified for the first time with droplet growth rates one and two orders of magnitude greater than during condensation–coalescence and direct condensation regimes, respectively. Findings presented here are of great importance for the effective design and implementation of LISs via surface structure endowing accurate droplet mobility and control for applications such as anti-icing, self-cleaning, water harvesting, and/or liquid repellent surfaces as well as for condensation heat transfer.

## Introduction

Enhanced droplet mobility is of intrinsic relevance to a wide range of industrial and everyday applications such as self-cleaning,^1^ drag reduction,^2^ anti-icing,^3^ water harvesting,^4^ macro-fouling prevention,^5^ and/or antireflection,^6^ among others. More specifically, in the past decades, considerable amount of research has been devoted to the design of surfaces that can favor the continuous nucleation, growth, and departure of the condensate in a dropwise condensation (DWC) fashion. The continuous shedding of droplets with sizes in the order of few millimeters via gravitational forces can be prompted by the implementation of a low surface energy coating, which renders the wettability of the surface hydrophobic.^7−11^ In addition to hydrophobic surfaces, nano-textured and hierarchical micro-/nano-textured surfaces further coated with a thin conformal hydrophobic layer, so-called superhydrophobic surfaces (SHSs), have also demonstrated to provide extremely low droplet–surface adhesion^12,13^ and enhanced condensation heat transfer performance.^14^ These are owed to the effective decrease of the liquid–solid binary interactions if droplets condense/sit on the micro-/nano-structures while air pockets are entrapped in between the structures with the consequent reduced adhesion to the surface.^15−18^ However, condensing droplets may also nucleate, grow, and coalesce within the micro-/nano-cavities, typically incurring in the partial wetting Wenzel regime with high adhesion and enhanced pinning.^19−21^ Pinning of the condensate induces the loss of efficient droplet shedding triggering undesirable flooding.^14,22,23^

To overcome such deficiency, lubricant-infused surfaces (LISs) have been proposed. The presence of a lubricant impregnated within the surface micro-/nano-structures impedes the direct nucleation and growth of the condensate within these, which overcomes the partial wetting Wenzel regime as well as undesired condensate adhesion or pinning. In addition, LISs offer virtually no-pinning, i.e., contact angle hysteresis *ca*. 2.5°, and droplet self-removal for surface inclinations angles below 5°.^24−26^ The excellent low adhesion reported on LISs is owed to the more affinity of the lubricant for the substrate underneath, typically a hydrophobic coating, hindering the intimate direct binary interactions between the solid surface and the condensing fluid, even for low surface tension fluids.^27,28^ In addition, the slippery Wenzel state on LISs has been recently reported overcoming the low droplet mobility associated with the partial wetting Wenzel state ensuing otherwise on nonlubricated hierarchical micro-/nano- or on micro-structured superhydrophobic surfaces.^29^ Besides the excellent properties reported above, LISs can achieve up to 100% greater heat transfer coefficients when compared to SHSs and/or to hydrophobic surfaces.^30^ Further, Preston *et al*. reported up to 400% enhancement on the heat transfer coefficient during hydrocarbon DWC on a LIS when compared to filmwise condensation (FWC).^31^ More recently, the occurrence of stable ethanol and hexane DWC with a 200% enhancement on the heat transfer coefficient on LISs when compared to FWC taking place on a traditional hydrophobic surface was demonstrated by Sett *et al*.^28^

The type of lubricant,^32,33^ the spacing between micro-structures,^25,29^ the presence of macro-patterns on the surface,^34^ the use of wettability gradient surfaces,^35^ and the size of the droplets,^36^ have all been reported to influence the droplet mobility on LISs and hence the shedding and/or roll-off angles. Nonetheless, an effective approach to enhance droplet mobility on LISs by minimizing the condensate–surface intimate interactions brought by the implementation of micro-structures without penalizing heat transfer is yet to be demonstrated. Hierarchical micro-/nano-structured LISs and solely nano-structured LISs were fabricated following facile and easy scalable etching and oxidation procedures and further lubricant impregnation. Fabricated surfaces are then investigated paying special attention to the dynamics of droplet shedding during condensation phase-change, i.e., condensate–coalescence–shedding regime. The greater droplet mobility on hierarchical micro-/nano-structured LISs is here reported for the first time and supported by our revisited force balance at the droplet–LIS triple contact line as well as via optical microscopy observations at the condensate–LIS interface looking through the condensing droplets. Furthermore, in spite of the additional heat transfer resistance imposed by the micro-structures, a similar heat transfer performance is achieved by the quicker condensate removal when compared to solely nano-structured LISs. We conclude on the greater mobility and greater shedding performance empowered by the implementation of the micro-structures on LISs when compared to solely nano-structured ones, which in turn can be tuned to encompass enhanced heat transfer, self-cleaning, and anti-icing performance. This strategy could be more specifically exploited in the accurate arrangement of textile fibers to minimize rain droplet adhesion and wicking or in the design of wings in planes or blades in wind turbines. Minimizing adhesion while maximizing the shedding of water or that of supercooled water droplets impacting on them, are of importance as otherwise these droplets would freeze on the surface modifying their structural and dynamic performance eventually causing accidents.

## Results and Discussion

### Design Rationale

**Figure 1:**
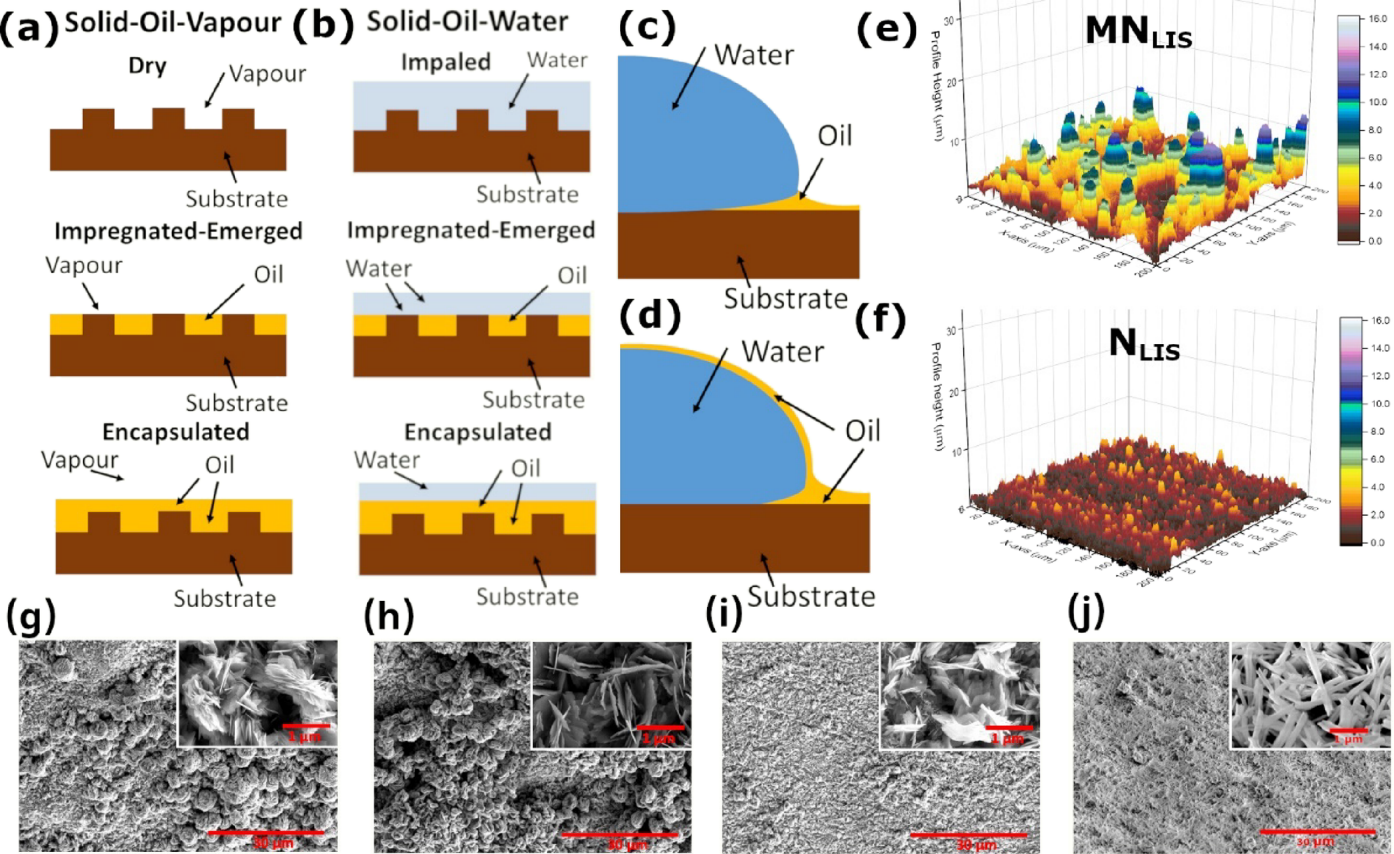
Schematic representation of the different thermodynamic configurations for (a) solid–lubricant–air and (b) solid–lubricant–water ternary systems. Schematic of a droplet on a LISs for (c) negative lubricant–water spreading coefficient or no encapsulation/cloaking *S*_ow_ < 0 and (d) positive lubricant–water spreading coefficient or encapsulation/cloaking *S*_ow_ > 0. 3D laser optical microscopy before impregnation for (e) micro-/nano-structured LIS or MN_LIS_ and (f) nano-structured LIS or N_LIS_. Color scale bar in (e) and (f) represents the surface structure height between (dark-brown) 0 μm and (light-gray) 16 μm. Scanning electron microscopy (SEM) images of samples (g) MN_LIS_, (h) mN_LIS_, (i) N_LIS_, and (j) n_LIS_. Additional surface structure characterization for all surfaces studied can be found in the Supporting Information Sections SI.1 and SI.2.

On the one hand, on a ternary system solid–lubricant–air, three different thermodynamically stable configurations are possible depending on the solid–lubricant, lubricant–air, and solid–air binary interactions,^25^ which are represented in Figure 1a. Typically, for low surface tension lubricants or complete wetting lubricants, i.e., contact angles between the lubricant and the solid surface of ca. 0°, lubricant impregnation/infusion within the structures of textured surfaces and encapsulation of the structures occur. Whereas for high surface tension ones, the lubricant may impregnate the micro- and the nano-structures while at the same time it is not energetically favorable for the lubricant to cover/encapsulate the tops of the micro-/nano-structures.^24,25^ In the case of high surface tension lubricants, far away from the lubricant, dry regions may be found due to the lack of complete wetting. On the other hand, on a ternary system solid-lubricant-water also three possible stable configurations exist aiming to minimize the overall surface energy of the system,^25^ which are represented in Figure 1b. A water droplet may displace the lubricant and contact the solid structures as in the impaled state and/or Wenzel state, may rest at the top of the solid structures with the lubricant impregnated in between structures, or may glide/sit over the lubricant as in the encapsulated state.^25,37^

In addition to the different wetting states reported above, upon droplet deposition on a LIS, the lubricant may or may not encapsulate/cloak the droplet depending on the lubricant–water spreading coefficient *S*_ow_ where *S*_ow_ = γ_la_ – γ_ol_ – γ_oa_^38^ with γ_la_, γ_ol_, and γ_oa_ as the binary liquid–air, lubricant/oil–liquid, and lubricant/oil–air interfacial tensions. For moderate and high surface energy lubricants, i.e., γ_ol_ + γ_oa_ > γ_la_, the spreading coefficient is typically negative and encapsulation/cloaking of the droplet by the lubricant does not occur as in Figure 1c. Whereas, for a low surface tension lubricant and a positive spreading coefficient, i.e., γ_ol_ + γ_oa_ < γ_la_, the lubricant does encapsulate/cloak the droplet as in Figure 1d. It is then clear that the intimate interactions between a droplet, the lubricant, and the surface are governed by the wetting configuration of the ternary systems: solid–lubricant–air and solid–lubricant–water. As such, during condensation phase change, the dynamics and mechanisms of droplet growth,^39^ coalescence,^40^ and more importantly the mobility of the condensing droplets^33,38^ will depend strongly on the two introduced ternary systems, which in turn are governed by the wettability^41^ and surface structure^25^ of the solid surface, the type^32^ and phase of lubricant,^42,43^ and the nature of the condensing fluid.^28,31,41^

For a droplet sitting on an inclined ideal smooth solid surface in ambient air, a force balance tangential to the surface can be established. A pinning force *F*_pin_ keeps the droplet attached to the surface, whereas a gravitational depinning force *F*_g_ pulls the droplet downward due to gravity. Then, for the droplet to move, *F*_g_ must overcome *F*_pin_ as in eq 1:^29,35,44,45^
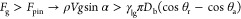
1where *V* is the droplet volume, *ρ* is the density of water, *α* is the inclination angle of the surface, *g* is the gravity acceleration, *θ*_a_ and *θ*_r_ are the advancing and receding droplet contact angles, and π*D*_b_ is the droplet wetting triple phase contact line with *D*_b_ as the base diameter, which during droplet growth, due to condensation, can be calculated as 2*R*sin*θ*_a_, where *R* is the droplet curvature radius. From eq 1, the force prompting the droplet motion *F*_g_ is a function of the droplet size, i.e., droplet volume, and of the surface inclination angle, whereas the force opposing to droplet shedding *F*_pin_ is proportional to the droplet base wetting perimenter π*D*_b_ and to the contact angle hysteresis: CAH ∼ cos*θ*_r_ – cos*θ*_a_. Based on eq 1, upon greater gravitational forces overcoming the pinning force, i.e., *F*_g_ – *F*_pin_ > 0, the excess of net force is then transformed into the droplet motion prompting shedding.^25,29,32,46^

### LIS Characterization

Two hierarchical micro-/nano-structured and two nano-structured copper oxide SHSs were fabricated. Big size and high density of the micro-structures (MN_LIS_) and small size and low density of micro-structures (mN_LIS_) were fabricated by varying the time and the temperature of the wet chemical etching procedure.^47^ Etched microstructured copper plates were further subjected to an oxidation step following the same temperature and dipping time for the nano-structures growth^48,49^ yielding MN_LIS_ and mN_LIS_. and N_LIS_.^50^ Moreover, two different nano-scale roughness samples, nano-structured blades (N_LIS_) and tube like nano-structures (n_LIS_), were fabricated following two different temperatures and dipping times during the oxidation procedure on smooth copper plates.^50,51^ Note that nano-structures decorating N_LIS_ were fabricated following the same oxidation procedure as for MN_LIS_ and mN_LIS_. In addition, functionalization of the surface by a hydrophobic coating prior impregnation was carried out as it is a necessary condition for inducing the more affinity of the lubricant to the surface than water.^2,41,52,53^Figure 1e,f highlights the presence and absence of micro-structures when comparing MN_LIS_ to N_LIS_. The complete details on the surface fabrication procedure can be found in the Materials and Methods Section and in the work of Zhang *et al..*(54) Meanwhile, further surface characterization via scanning electron microscopy (SEM) and 3D laser optical microscopy for all four LISs before lubricant impregnation can be found in the Supporting Information Sections SI.1 and SI.2 and Figures SI.1–SI.3. Figures SI.1–SI.3 highlight the greater size and density of the micro-structures decorating MN_LIS_ compared to mN_LIS_ and the absence of micro-structures on N_LIS_ and n_LIS_. From the 2D laser optical microscopy profiles included in Figures SI.2 and SI.3, the micro-structure solid fraction, Ω, for MN_LIS_ and mN_LIS_ is calculated as 0.305 and 0.196, respectively (see Figures SI.2 and SI.3 in the Supporting Information).

Two different Krytox General-Purpose Lubricant 103 and 107 from DuPont (USA), henceforth referred to as GPL103 and GPL107, respectively, were used. The surface tension of the lubricant in air *γ*_oa_ and that of the lubricant in water γ_ol_ were also measured in a custom built goniometer and further analyzed by an ImageJ plugin^55^ as 16.1 ± 0.2 and 53.1 ± 1.8 mN/m, respectively, for GPL103, and 17.4 ± 0.3 and 54.3 ± 1.4 mN/m for GPL107. It is worth noting that *γ*_oa_ and γ_ol_ reported here are in close agreement with values reported earlier in the literature.^40,56^ Further schematics and procedure followed for the characterization of the *γ*_oa_ and the *γ*_ol_ can be found in the Supporting Information SI.5. Next, the spreading coefficient *S*_ow_ for GPL103 and for GPL107 in water is estimated as *S*_ow_GPL103_ = 3.6 mN/m and *S*_ow_GPL107_ = 1.1 mN/m, respectively, and hence the lubricant cloaks the condensing droplets, i.e., *S*_ow_ > 0 mN/m, as represented in Figure 1d.^25,52^^40^ The critical thickness of the cloaking film, *δ*_lubricant_, was estimated as  where *A*_H_ is the Hamaker constant, ∼10^–18^ J, *R*_c_ is the droplet radius of curvature, ∼1 mm, and *γ*_la_ is the water–air interface approximated as *γ*_lo_ + *γ*_oa_.^40,54^ Then, *δ*_lubricant_GPL103_ = 73 nm and *δ*_lubricant_GPL107_ = 72 nm, which are also in agreement with *δ*_lubricant_ ≈ 100 nm earlier reported in the literature.^40,54^

Next, to further characterize the different solid–lubricant–air and solid–lubricant–water thermodynamic configurations, the contact angle of the two lubricants on the different SHSs are measured. In the case of GPL103, its equilibrium contact angle on all four SHSs in air before impregnation, *θ*_os(a)_, was found to be independent of the surface structure underneath the lubricant equals 8° ± 3°. Whereas in the case of GPL107, the equilibrium contact angle on the micro-structured MN SHS was *θ*_os(a)_ ≈ 18° ± 3° and on the nano-structured N SHS *θ*_os(a)_ ≈ 11° ± 3° (see Supporting Information SI.3 for more details on the surface–lubricant characterization). The low finite *θ*_os(a)_ below the critical angle for hemiwicking *θ*_c_ independently of the type of lubricant studied (*θ*_c_ ≈ 30°) evidences that the solid–lubricant–air ternary system on all substrates reported in this work behave in the impregnated-emerged state as represented in Figure 1b with the tops of the structures exposed to the ambient or to the condensate. The critical angle for hemiwicking *θ*_c_ is calculated as  where *f* is the solid fraction and *φ* is the roughness factor^57^ (see Supporting Information SI.3 for more details on these calculations). In the impregnated-emerged mentioned state, the lubricant impregnates the micro- and the nano-structures; however, it is not thermodynamically favorable for the lubricant to cover/encapsulate the top of the structures and these are then exposed to the ambient and to the water vapor.^25,58^ Hence, during condensation phase-change, favorable heterogeneous nucleation shall take place at the top of the nano-structures and the ternary system solid–lubricant–water behaves then in the impregnated-emerged state represented in Figure 1b. In such an impregnated/emerged state, the condensate intimately interacts solely with the top of the nano-structures, while the lubricant confined within the structures hinders further interactions between the condensate and the solid surface.

One of the main features of LISs is the extremely low CAH reported in the order of few degrees.^24,25,29^ Hence, macro-scopic advancing and receding contact angles, *θ*_a_ and *θ*_r_, for water on the different LISs were also measured in a custom-built goniometer and analyzed with ImageJ.^59^ The nano-structure solid fraction *f* was estimated from the Cassie–Baxter equation before impregnation as *f* = (cos *θ*_a_ + 1)/(cos *θ*_a_^flat^ + 1),^60^ where *θ*_a_ and *θ*_a_^flat^ are the advancing contact angle on the SHS and on the flat hydrophobic surface, respectively, with *θ*_a_^flat^ = 112° ± 2°. Table 1 summarizes then the data on the structural characterization of the SHSs before impregnation and on the *θ*_a_, *θ*_r_, and CAH on the different LISs after impregnation, which are in agreement with earlier results on similar fabricated substrates.^50^ We highlight here that the presence or absence of micro-structures underneath the infused lubricant and the type of lubricant did not influence considerably the macroscopic *θ*_a_ and *θ*_r_ and hence the contact angle hysteresis (CAH = *θ*_a_ – *θ*_r_), which is ca. 3° on all four LISs. Complete characterization of the hierarchical micro-/nano- and the solely nano-structured surfaces including advancing and receding contact angles are included in Table 1 and in the Supporting Information Section SI.4 and Table SI.II. Looking closely at eq 1, the similar CAH exerted ca. 3° independently of the LISs studied here suggests that that *F*_pin_ is only a function of the *D*_b_, i.e., the droplet size.

**Table 1:** Substrate Structural Characterization of MN_LIS_, mN_LIS_, N_LIS_, and n_LIS_^a^

	**MN**_**LIS**_	**mN**_**LIS**_	**N**_**LIS**_	**n**_**LIS**_
*S*_RMS_ (μm)	2.1	1.4	0.8	0.5
*f*	0.11	0.11	0.11	0.10
Ω	0.31	0.20		
CAH__SHS_	<1°	<1°	<1°	<1°
*θ*_a_GPL103_ (deg)	114° ± 2°	118° ± 2°	117° ± 2°	118° ± 2°
*θ*_r_GPL103_ (deg)	111° ± 4°	115° ± 4°	114° ± 2°	115° ± 3°
CAH_GPL103_	3° ± 2°	3° ± 2°	3° ± 3°	3° ± 3°
*θ*_a_GPL107_ (deg)	113° ± 2°	114° ± 3°	114° ± 2°	114° ± 3°
*θ*_r_GPL107_ (deg)	110° ± 2°	110° ± 2°	110° ± 2°	111° ± 3°
CAH_GPL107_	3° ± 2°	4° ± 3°	4° ± 2°	3° ± 3°

a Surface roughness *S*_RMS_(μm), microstructure solid fraction Ω (−), nano-structures solid fraction *f* (−), and contact angle hysteresis (CAH_*_SHS*_) prior to impregnation; surface wettability characterization as advancing contact angle *θ*_a_ (deg), receding contact angle *θ*_r_ (deg), and contact angle hysteresis CAH (deg), on MN_LIS_, mN_LIS_, N_LIS_, and n_LIS_ impregnated with GPL103 and GPL107. Contact angle measurements report the average and standard deviation for at least five different independent measurements.

### Hypothesis Validation: Experimental Observations of Condensation Phase-Change

**Figure 2:**
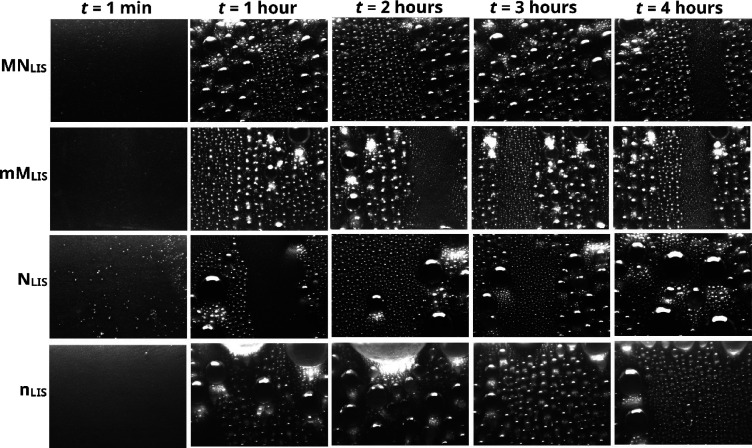
Characteristic condensation phase-change behavior for a period of 4 h on the different MN_LIS_, mN_LIS_, N_LIS_, and n_LIS_ infused with GPL103 at *t* = 1 min and at *t* = 1, 2, 3, and 4 h. The width of each frame is approximately 5 mm, which can be used as scale bar.

Next, we present experimental observations of condensation phase-change in time for a period of 4 h for the different hierarchical micro-/nano-structured LISs when compared to solely nano-structured LISs in Figure 2:

From Figure 2, after 1 min, very small droplets nucleate on the surface, which can be barely noticed as per the rather low magnification of the snapshots. As time progresses, small droplets grow bigger via direct condensation until they reach the effective transition radius at which thereafter droplets grow via both direct condensation as well as coalescence with neighboring droplets. Once the droplets are big enough to overcome the adhesion to the surface via gravitational forces, shedding of the droplets take places, which refreshes the condensing surface for further renucleation, growth, and shedding, which is characteristic of high heat transfer.^14,39,51^ From Figure 2, the shedding of droplets at different intervals of time is apparent as per the regions of the surface in the form of vertical stripes showing different droplet size densities. These vertical stripes are formed upon a droplet shedding event and as such contain much smaller droplets than their surroundings as it can be clearly appreciated for almost all intervals of time presented in Figure 2. A clear example is N_LIS_ after 1 h of condensation where a shedding event just took place. In addition, to highlight from Figure 2 is the rather larger droplets present at any given instance of time observed on nano-structured N_LIS_ and n_LIS_ when compared to MN_LIS_ and mN_LIS_. The reasons for the greater size of the observed droplets along with the different shedding behavior depending on the LISs are presented next. This was additionally demonstrated by the droplet number density figures reported in Maeda *et al.* where smaller sized droplets are present over the condensation times analyzed on micro-structured MN_LIS_ and mN_LIS_.^51^

**Figure 3:**
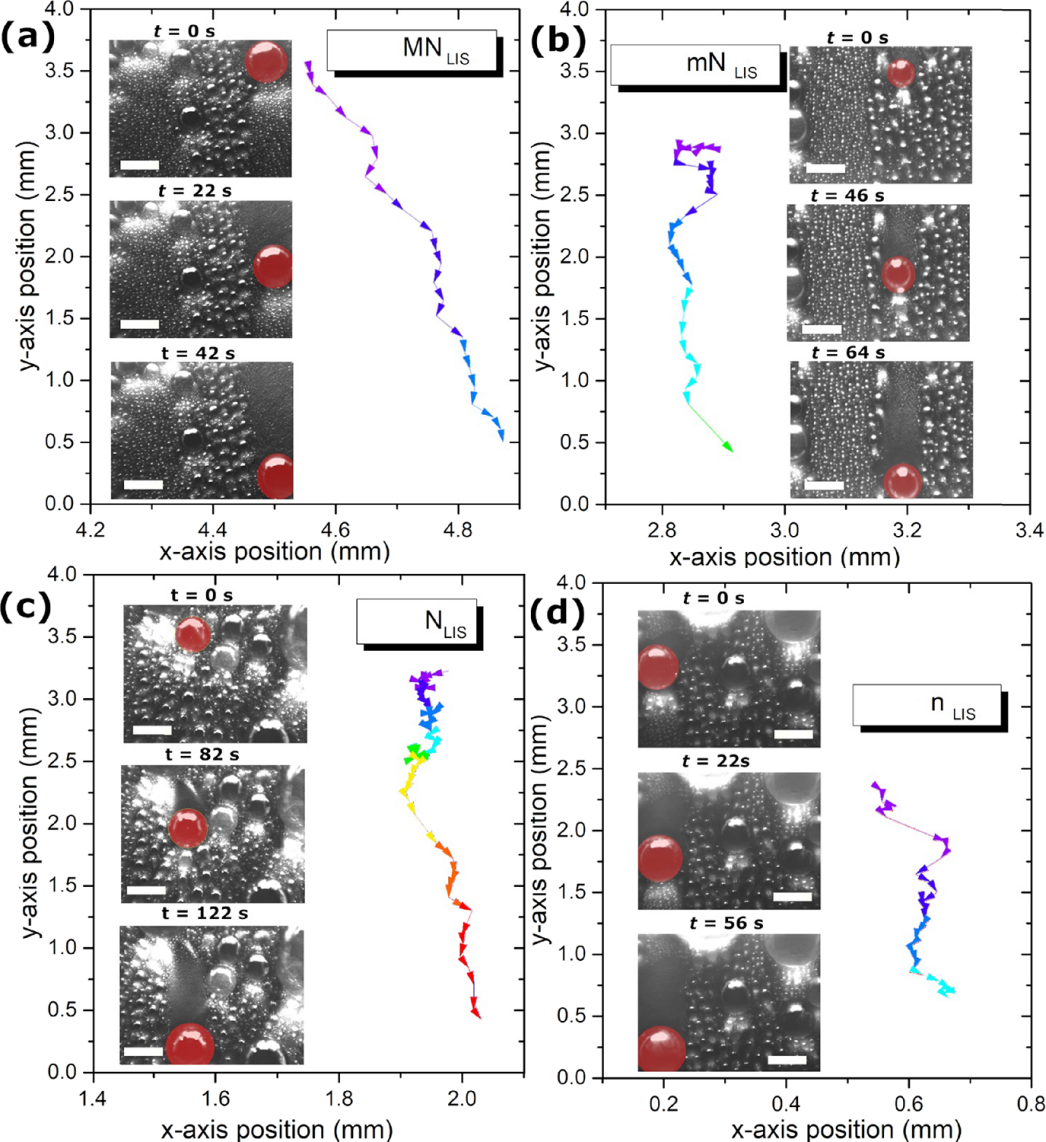
Characteristic droplet motion during shedding as droplet trajectory and distance traveled during shedding on GPL103 LISs for (a) MN_LIS_, (b) mN_LIS_, (c) N_LIS_, and (d) n_LIS_. False red color has been applied to readily identify the shedding droplets. Scale bar within snapshots is 1 mm, while (0, 0) position represents the bottom left corner of the snapshot. Each vector represents the motion within 2 s. In addition, color code has been applied to illustrate the time as a rainbow balanced scale from purple to red for an approximate 10 s per color.

**Figure 4:**
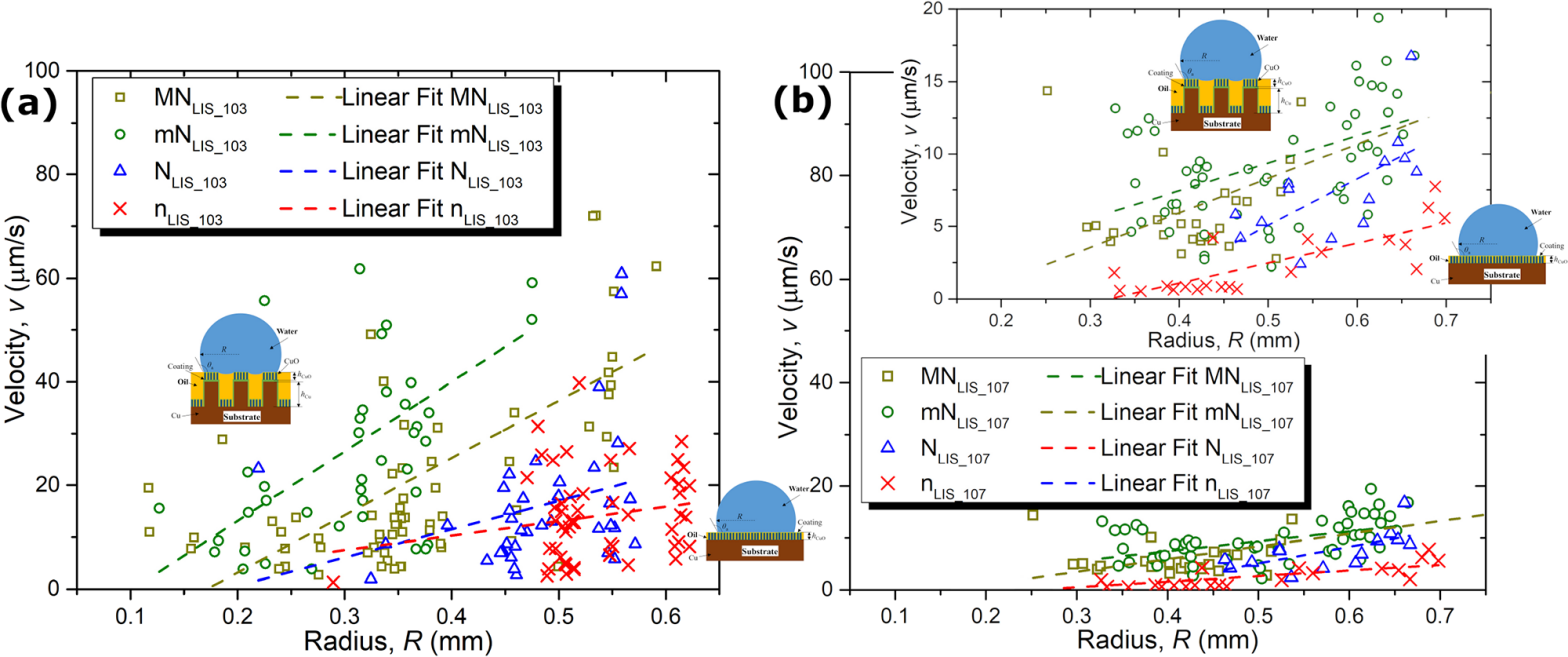
Droplet velocity, *v* (μm/s), versus droplet curvature radius, *R* (mm), on micro-/nano-structued (dark yellow squares) MN_LIS_ and (green circles) mN_LIS_, and nano-structured (blue up-triangles) N_LIS_, and (red crosses) n_LIS_ for LISs impregnated with (a) GPL103 and (b) GPL107, from at least three different droplet shedding events. Inset in (b) is an enlarged snapshot of (b) showing the velocity range between 0 and 20 μm/s. In both figures (a) and (b), only droplet motion events where the change in droplet radius due to condensation and/or coalescence is <1% are represented. Linear fittings are included to illustrate the trend. Velocity error is estimated as ±10%, whereas droplet radius error is ±0.02 mm. Insets include schematics of a droplet on (top-left) hierarchical micro-/nano-structured LISs and (bottom-right) nano-structured LISs.

Experimental results during condensation phase-change demonstrating the lower adhesion along with the greater droplet mobility in the presence of hierarchical micro-/nano-structured LISs when compared to solely nano-structured LISs are now introduced. To support these findings, we focus on the mobility and shedding performance of the condensing droplets at the macroscale, i.e., ability to refresh the surface for droplet renucleation and growth. Figure 3 includes characteristic snapshots extracted during a representative droplet shedding event during macroscopic observations of condensation phase-change, which include the trajectory and distance traveled every 2 s on all four LISs impregnated with GPL103. Note that Figure 3 just includes a representative case while Figure 4 includes further analysis of at least three different droplet shedding events, which will be introduced and discussed later.

Figure 3 reports clear differences when comparing the size and the motion of the shedding droplets depending on the substrate studied. On both nano-structured LISs (Figure 3c,d) droplets with diameters above 1 mm are required for droplet shedding to ensue, whereas on hierarchical micro-/nano-structured LISs (Figure 3a,b) droplets with diameters near or below 1 mm are mobile and able to shed from the surface. The smaller nature of the shedding droplets with sizes in the submillimeter range in addition to the greater distances covered within less time on MN_LIS_ and mN_LIS_, i.e., greater droplet mobility, when compared to N_LIS_ and n_LIS_, are here highlighted.

Next, we present further data analysis on the droplet mobility and shedding performance for all four LISs. From experimental observations, the droplet velocity *v* (μm/s) versus droplet curvature radius *R* (mm), over a 4 h condensation phase-change period, is extracted by tracking the position of the droplet center of mass and the size of mobile droplets in time using ImageJ (more details on the data analysis procedure can be found in the Supporting Information SI.7). The droplet velocity *v* (μm/s) versus droplet curvature radius *R* (mm) values for GPL103 LISs: MN_LIS_103_, mN_LIS_103_, N_LIS_103_, and n_LIS_103_, and for GPL107 LISs: MN_LIS_107_, mN_LIS_107_, N_LIS_107_, and n_LIS_107_, are then represented in Figure 4a and Figure 4b, respectively. We note here that during dynamic condensation, droplet motion can be triggered by gravity and/or by coalescence with neighboring droplets.^33,38^ Nonetheless, to primarily account for the droplet motion induced mainly by gravity, so to minimize and rule out the effect of coalescence and/or sweeping, Figure 4 only reports droplet velocities where the change in droplet radius due to condensation and/or coalescence is <1%.

From Figure 4, the expected greater droplet velocities as the size of the droplets increase independently of the LIS studied are demonstrated, which follows the force balance introduced in eq 1. Nonetheless, differences on the size and on the velocities of the shedding droplets are readily identified when comparing the eight different LISs studied. On solely nano-structured N_LIS_ and n_LIS_, i.e., absence of micro-structures, for the droplet motion to ensue, most of the droplet raidii are found to be approximately 0.5 mm or above, i.e., 1 mm in diameter. Whereas, on hierarchical MN_LIS_ and mN_LIS_, droplets with diameters in the submillimeter range, i.e., below 1 mm, are actually found to be mobile as previously highlighted in Figure 3a and Figure 3b. Furthermroe, when comparing droplets of similar size, greater droplet velocities are reported on hierarchical micro-/nano-structured LISs (MN_LIS_ and mN_LIS_) than on nano-structured ones (N_LIS_ and n_LIS_). Moreover, the expected greater mobility of droplets on LISs impregnated with a low viscosity lubricant (GPL103) when compared to a high viscosity one (GPL107) as in the work of Daniel *et al.*(32) is additionally supported when comparing Figure 4a,b. Last, we remind the reader here that droplet shedding velocities reported in Figure 4 are primarily attributed to gravitational effects opposite to other works where coalescence with neighboring droplets inducing sweeping was also accounted for.

### Revisited Force Balance

When looking into eq 1, on the one hand, for all LISs studied *F*_g_ scales with *R*^3^ while *F*_pin_ scales with *D*_b_ or with *r*_b_ both of them proportional to *R*, which directly supports the greater shedding velocities as the droplet size increases reported in Figure 4. On the other hand, when looking into *F*_pin_, the relatively similar CAH reported of ca. 3° indepdently of the LIS studied suggests that for the same droplet size *F*_pin_ shall be the same and droplets should shed from the surface for a similar *F*_g_, i.e., for a similar droplet size. Nonetheless, when looking into the sizes of the shedding droplets, clear differences are found depending on the surface structure and the lubricant viscosity of the LIS. Tables 2 and 3 provide quantification on the average and standard deviation of the droplet shedding behavior from at least three different droplet shedding events for the different LISs impregnated with GPL103 and GPL107 LISs, respectively. Note that over the 4 h duration of the experiments up to 14 droplets shed off the surface; however, only three droplets were fully analyzed as other droplets may have been partially outside the field of view or undergone major coalescence events. The average radius, *R̅* (mm), volume, *V̅* (μL), and velocity, *v̅* (μm/s), were calculated from the data reported in Figure 4, from at least three different droplet shedding events, while the gravitational force, *F*_g_ (μN), and pinning force, *F*_pin_LIS_ (μN), have been calculated by making use of eq 1 and the average curvature radius, *R̅* (mm), volume, *V̅* (μL), and droplet velocity *V̅* (μm/s), reported.

**Table 2:** Average and Standard Deviation of Droplet Shedding Radius or Curvature Radius, *R̅* (mm), Volume, *V̅* (μL), and Droplet Velocity *v̅* (μm/s), Calculated from the Data Reported in Figure 4 from at Least Three Different Droplet Shedding Events and Gravitational Force, *F*_g_ (μN), and Pinning Force, *F*_pin–LIS_ (μN), Calculated by Making Use of Eq 1 and the Average Curvature Radius, *R̅* (mm), Volume, *V̅* (μL), and Droplet Velocity *v̅* (μm/s), Reported Here for MN_LIS_, mN_LIS_, N_LIS_, and n_LIS_ Impregnated with GPL103

	*R̅***(mm)**	*V̅***(μL)**	*v̅* (μm/s)	***F*_g_(μN)**		***F*_pin–LIS_(μN)**
MN_LIS_GPL103_	0.37 ± 0.11	0.21 ± 0.18	21.5 ± 22.5	2.1 ± 1.8	<	7.3 ± 2.3
mN_LIS_GPL103_	0.31 ± 0.07	0.12 ± 0.08	27.9 ± 20.1	1.2 ± 0.8	<	5.9 ± 1.5
N_LIS_GPL103_	0.48 ± 0.07	0.39 ± 0.15	15.8 ± 12.7	4.1 ± 1.5	<	9.0 ± 1.4
n_LIS_GPL103_	0.54 ± 0.06	0.57 ± 0.17	14.4 ± 8.2	5.6 ± 1.7	<	10.0 ± 1.1

**Table 3:** Average and Standard Deviation of Droplet Shedding Radius or Curvature Radius, *R̅* (mm), Volume, *V̅* (μL), and Droplet Velocity *v̅* (μm/s), Calculated from the Data Reported in Figure 4 from at Least Three Different Droplet Shedding Events and Gravitational Force, *F*_g_ (μN), and Pinning Force, *F*_pin–LIS_ (μN), Calculated by Making Use of Eq 1 and the Average Curvature Radius, *R̅* (mm), Volume, *V̅* (μL), and Droplet Velocity *v̅* (μm/s), Reported Here for MN_LIS_, MN_LIS_, N_LIS_, and N_LIS_ Impregnated with GPL107

	*R̅***(mm)**	***V̅* (μL)**	***v*®** (μm/s)	***F*_g_(μN)**		***F*_pin–LIS_(μN)**
MN_LIS_GPL107_	0.47 ± 0.15	0.47 ± 0.52	7.6 ± 5.3	4.6 ± 5.1	<	9.6 ± 3.1
mN_LIS_GPL107_	0.50 ± 0.1	0.49 ± 0.28	9.5 ± 4.0	4.8 ± 2.7	<	13.5 ± 2.8
N_LIS_GPL107_	0.58 ± 0.07	0.71 ± 0.25	7.5 ± 3.5	7.0 ± 2.4	<	15.4 ± 1.9
n_LIS_GPL107_	0.50 ± 0.12	0.50 ± 0.35	2.5 ± 2.1	4.9 ± 3.5	<	10.0 ± 2.5

From Tables 2 and 3, the *F*_pin_LIS_ > *F*_g_ reported anticipates that droplets should not shed from the surface; hence, eq 1 fails to accurately account for the experimental observations of droplet shedding during dynamic condensation reported on all the LISs studied. Note that *F*_pin_LIS_> *F*_g_ prevails even if assuming a 1° of CAH for the calculations of *F*_pin_LIS_, which does not support the droplet shedding observations reported. CAH below 1° has been reported on the superhydrophobic surface prior lubricant impregnation, i.e., upon inteaction of the liquid droplets with the structures tops. While upon impregnation the CAH increases up to ca. 3° ± 1° as a consequence of the greater droplet footprint with the consequent greater number of finite interactions with the structures’ tops and presumabily owed to the oil viscosity hindering the contact line motion. It is then safe to assume that CAH values reported on our LISs are governed by the droplet itnteractions with the structures’ tops. Hence, it becomes apparent then that the force balance analysis proposed in eq 1(29,44,45) needs to be revisited in order to accurately predict *F*_pin_LIS_, which is an important parameter required for the accurate design of LISs with enhanced mobility.

**Figure 5:**
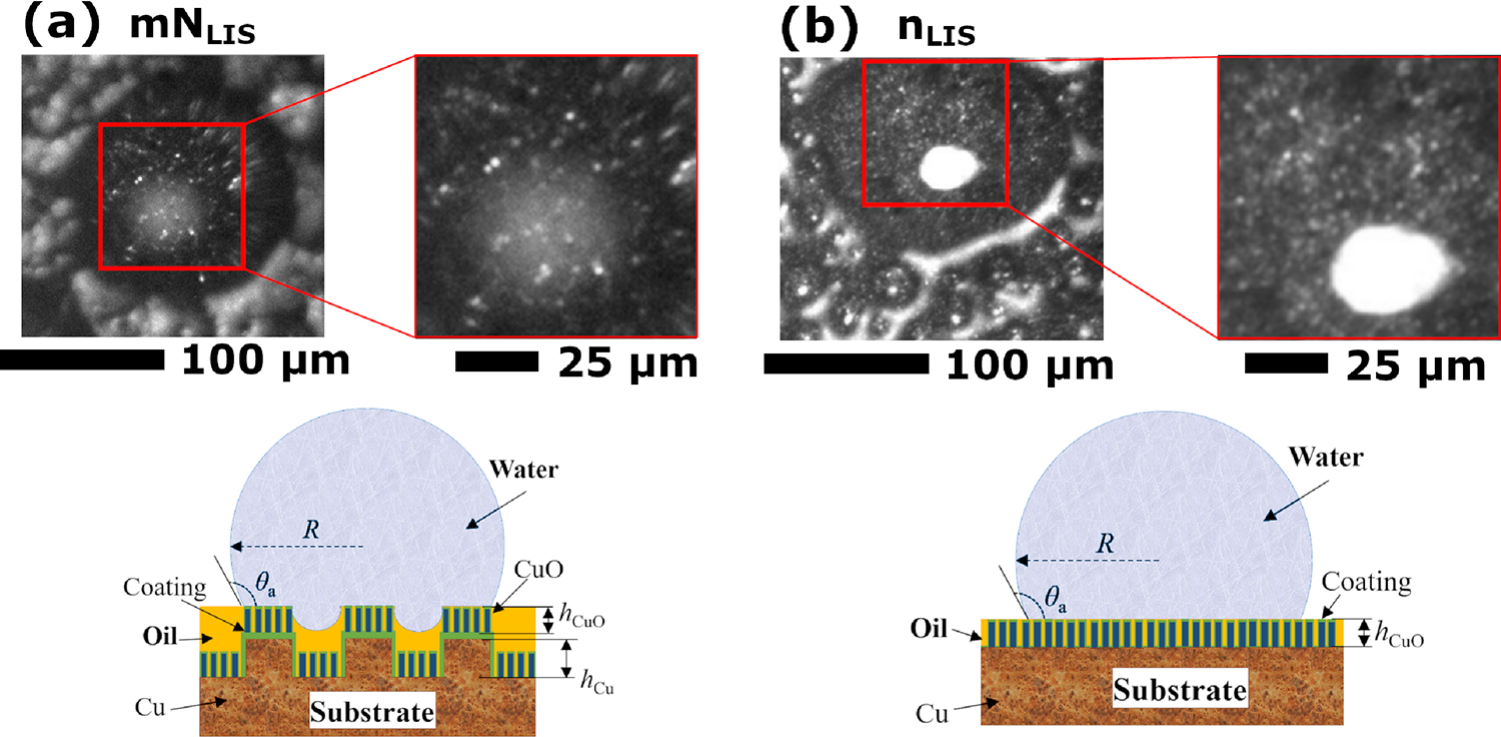
High-resolution zoom lens optical microscopy observations through condensing droplets at the droplet–LIS interface and schematic representation of the cross section of a droplet sitting on (a) hierarchical micro-/nano-structured MN_LIS_ and (b) solely nano-structured n_LIS_. Scale bars are 100 and 25 μm. Note that the large white area in the order of tens of micrometers in size is a consequence of the light reflection at the droplet liquid–gas interface rather than that at the droplet–LIS interface.

To further characterize the pinning mechanisms taking place between the condensing droplets and the different LISs we must closely look at the intimate interactions at the LISs structures.^61,62^ Based on the thermodynamic criteria established earlier, the lubricant typically impregnates the micro- and the nano-structures while the tops of the nano-structures on N_LIS_ and n_LIS_ and the top of the nano-structures atop of the micro-structures only on MN_LIS_ and mN_LIS_, emerge from the lubricant and are exposed to the ambient and the vapor.^25^ Thus, water vapor nucleates and condenses at the top of the nano-structures and droplets then grow following the impregnated/emerged solid-lubricant-water ternary system state represented in Figure 1b. To further demonstrate the interactions between the condensing droplets and the LISs, close experimental observations of the intimate binary interactions taking place right at the droplet-LISs interface are carried out and presented in Figure 5. The droplet–LIS interactions can be then characterized by the different brightness retrieved, where dark/black pixels represent the droplet-lubricant interactions while white/bright pixels are attributed to the droplet interactions with the emerging structures. In addition, the expected schematic representation of a water droplet sitting on a micro-/nano-structured LIS MN_LIS_ and on a solely nano-structured LIS N_LIS_ are also included within Figure 5a and Figure 5b, respectively. We note here that although copper micro- and nano-structures display a black color, which coupled to the different orientation of the structures, have a trapping light effect with up to 95% of the total incident visible light; at least 5% of the incident light maybe reflected in the normal direction to the surface, which is parallel to the incident light.^63^ Hence, the different contrast observed may be a consequence of the top of the structures reflecting 5% of the incident light while most of the incident light is trapped due to the black color as well as the different orientation of the structures and the multiple reflections within them surrounding the nano-structures tops.

When looking at Figure 5b, the greater/denser population of white/bright sharp pixels on nano-structured LISs N_LIS_ and n_LIS_ demonstrates the greater droplet/liquid–structure/surface interactions, which is also exemplified in the schematics. In this configuration, the base area of the condensing droplets is in contact with the top of the nano-structures and with the impregnated lubricant in between the nano-structures. Whereas, on hierarchical micro-/nano-structured MN_LIS_ and mN_LIS_, the larger area of black/dark pixels represents the more predominant interactions between the droplet and the lubricant. This is a consequence of the larger amount of lubricant impregnated between the micro-structures interacting with the droplet as only the nano-structures atop of the micro-structures directly interact with the liquid/droplet. Thus, the droplet–solid intimate interactions are greatly reduced as a consequence of the introduction of the micro-structures. Then, the effective droplet–LIS interactions must be proportional to the solid fraction, which in turn for hierarchical LIS is proportional to the micro-structure solid fraction as condensing droplets will only interact with the nano-structures present at the uppermost level of the hierarchical roughness.^64^ The effective solid fraction area is then defined as *φ* and hence *F*_pin-LIS_ scales to the effective solid faction along the perimeter of the contact line equals , and eq 1 becomes eq 2 as follows:
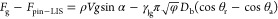
2

Note that eq 2 predicts the onset of the shedding while in order to describe the droplet motion the reader is referred to the work of Smith *et al.* where the driving forces scale with the capillary number *Ca* = *η*_o_*v*/*γ*_ol_ with *η*_o_ as the viscosity of the lubricant oil.^25,36^

Hence, in the case of N_LIS_ and n_LIS_, the effective pinning/structure fraction available to interact with the condensing droplets must be approximated to the solid fraction of the nano-structures *f* included in Table 1, i.e., *φ* = *φ*_nano_ = *f*. Whereas, for MN_LIS_ and mN_LIS_, the effective pinned fraction for multiple hierarchies *φ*_micro & nano_ is proportional to the nano-structures solid fraction *f* times the solid fraction of micro-structures Ω as *φ* = *φ*_micro & nano_ = Ω × *f*.^64^ Then, for MN_LIS_ and mN_LIS_, the micro-structures solid fraction Ω is calculated from 3D laser optical microscopy profiles as 0.305 and 0.196, respectively (see Figures SI.2 and SI.3 in the Supporting Information). Meanwhile, the nano-structure solid fraction *f* was estimated from the Cassie–Baxter equation before impregnation as *f* = (cos *θ*_a_ + 1)/(cos *θ*_a_^flat^ + 1).^60^ From all experimental results reported in Figure 4, we can then recalculate, making use of eq 2, the average and standard deviation of the droplet shedding radius *R̅*, droplet volume *V̅*, and shedding velocity *v̅*, and then the corresponding gravitational force *F*_g_, and pinning force *F*_pin–LIS_. Revisited calculations now account for the different droplet–surface interactions depending on the structure of the LIS studied, which are included in Tables 4 and 5 for GPL103 and GPL107, respectively.

**Table 4:** Average and Standard Deviation of Droplet Shedding Radius or Curvature Radius, *R̅* (mm), Volume, *V̅* (μL), and Droplet Velocity *v̅* (μm/s), Calculated From the Data Reported in Figure 4 from at Least Three Different Droplet Shedding Events and Gravitational Force, *F*_g_ (μN), and Pinning Force, *F*_pin–LIS_ (μN), Calculated by Making Use of Eq 2 and the Average Curvature Radius, *R̅* (mm), Volume, **v̅** (μL), and Droplet Velocity *V̅* (μm/s), Reported Here for MN_LIS_, MN_LIS_, N_LIS_, and N_LIS_ Impregnated with GPL103

	***R̅* (mm)**	*V̅***(μL)**	***v*®** (μm/s)	***F*_g_(μN)**		***F*_pin–LIS_(μN)**
MN_LIS_GPL103_	0.37 ± 0.11	0.21 ± 0.18	21.5 ± 22.5	2.1 ± 1.8	>	1.3 ± 0.4
mN_LIS_GPL103_	0.31 ± 0.07	0.12 ± 0.08	27.9 ± 20.1	1.2 ± 0.8	>	0.9 ± 0.2
N_LIS_GPL103_	0.48 ± 0.07	0.39 ± 0.15	15.8 ± 12.7	4.1 ± 1.5	>	3.0 ± 0.5
n_LIS_GPL103_	0.54 ± 0.06	0.57 ± 0.17	14.4 ± 8.2	5.6 ± 1.7	>	3.2 ± 0.4

**Table 5:** Average and Standard Deviation of Droplet Shedding Radius or Curvature Radius, **R̅** (mm), Volume, **V̅** (μL), and Droplet Velocity *v̅* (μm/s), Calculated from the Data Reported in Figure 4 from at Least Three Different Droplet Shedding Events and Gravitational Force, *F*_g_ (μN), and Pinning Force, *F*_pin–LIS_ (μN), Calculated by Making Use of Eq 2 and the Average Curvature Radius, **R̅** (mm), Volume, *V̅* (μL), and Droplet Velocity *v̅* (μm/s), Reported Here for MN_LIS_, MN_LIS_, N_LIS_, and N_LIS_ Impregnated with GPL107

	***R̅* (mm)**	*V̅***(μL)**	***v*®** (μm/s)	***F*_g_(μN)**		***F*_pin–LIS_(μN)**
MN_LIS_GPL107_	0.47 ± 0.15	0.47 ± 0.52	7.6 ± 5.3	4.6 ± 5.1	>	1.8 ± 0.6
mN_LIS_GPL107_	0.50 ± 0.1	0.49 ± 0.28	9.5 ± 4.0	4.8 ± 2.7	>	2.0 ± 0.4
N_LIS_GPL107_	0.58 ± 0.07	0.71 ± 0.25	7.5 ± 3.5	7.0 ± 2.4	>	5.1 ± 0.6
n_LIS_GPL107_	0.50 ± 0.12	0.50 ± 0.35	2.5 ± 2.1	4.9 ± 3.5	>	3.2 ± 0.9

By making use of eq 2, the pinning force, accounting for the effective pinned fraction of the triple phase contact line depending on the surface structure underneath the condensing droplets, *F*_pin-LIS_ is now smaller than gravitational forces *F*_g_ for both LISs impregnated with GPL103 and GPL107 as it can be seen in Tables 4 and 5. Hence, *F*_g_ > *F*_pin-LIS_ calculated by eq 2, unlike eq 1, does now demonstrate the observed onset of droplet shedding reported in Figure 3 and Figure 4. The new force balance does now agree both qualitatively and quantitatively with the sizes of the shedding droplets, which additionally anticipates and supports the feasibility of submillimeter droplets shedding from the surface in the presence of micro-/nano-structured LISs, i.e., MN_LIS_ and mN_LIS_, when compared to nano-structured N_LIS_ and n_LIS_ and to smooth hydrophobic surfaces.^8,38^ Besides the smaller nature of the shedding droplets in the submillimetre range reported on micro-/nano-structured MN_LIS_ and mN_LIS_, greater velocities for similar sized droplets are reported on hierarchical micro-/nano-structured MN_LIS_ and mN_LIS_ when compared to nano-structured N_LIS_ and n_LIS_. These findings are both supported via experimental observations and via the force balance accounting for the effective reduction of the condensate–LIS interactions by the introduction of the micro-features. In addition, findings are further demonstrated for both GPL103 and GPL107 impregnating lubricants on the same structured LIS; though in the case of the high viscosity oils, lower velocities of the shedding droplets are reported as expected.^25,32,36^ Results presented so far convey that nano-structured LISs are not slippery enough and droplet shedding is favored in the presence of micro-structures, i.e., hierarchical micro-/nano-structured LISs.

We must note here that although the presence of structures introduces an additional heat transfer resistance both through the micro-structures and the lubricant in between the structures,^51^ the better droplet shedding performance could in turn enhance the condensate removal and eventually the heat transfer performance.^8,33,65^ In order to provide further insights on the effect of enhanced droplet shedding on the heat transfer performance, the next subsection addresses the dynamics of droplet growth looking at individual droplets on the different LISs studied.

### Droplet Growth

When looking at the individual droplet growth, three distinctive regimes, namely, direct condensation, condensation-coalescence, and condensation-coalescence-shedding, have been identified and their different dynamics quantified. In the presence and absence of coalescence, droplet growth typically scales as ⟨*D*⟩ ∝ A*t*^μ^ where ⟨*D*⟩ is the average droplet diameter, A is a proportionality constant, *t* is time, and μ is the droplet growth power law exponent, which ranges between 0 and 1.^65−67^ While a clear droplet dynamics growth distinction between direct condensation and condensation–coalescence was proposed in the seminal work of Beysens and Knobler where the radius of isolated droplets was found to grow proportional to *t*^0.23^ during direct condensation and proportional to *t*^0.75^ during condensation–coalescence.^66^

First, we estimate the droplet growth performance during direct condensation and condensation-coalescence for droplets smaller than 200 μm via optical microscopy, while macroscopic observations were coupled for the characterization of the condensation–coalescence and the condensation–coalescence–shedding regime for droplets between 200 μm and their shedding sizes. Note that all droplet growth values reported in this subsection have been estimated from at least 3 different droplet growth events within the span of 4 h. The rather small standard deviation on the droplet growth during direct condensation and condensation–coalescence as well as that of the droplet shedding sizes reported imply that the droplet–LIS interactions do not significantly change and the lubricant can be assumed to be stable over the duration of the experimental observations. More details on the optical microscopy and macroscopic observation setup adopted can be found on the Materials and Methods Section, in the Supporting Information SI.6 and SI.7, and in ref (51).^51^ During these observations, the droplet size in time was tracked for up to three different droplets growing and shedding from the surface and the different droplet growth scaling ⟨*D*⟩ ∝ A*t*^μ^ was then provided based on their averages. A summary of the different proportionality constant and droplet growth power exponent for the various LISs impregnated with GPL103 under the different droplet growth regimes envisaged are reported below in Table 6:

**Table 6:** Average and Standard Deviation of Droplet Growth ⟨*D*⟩ as A*t*^μ^ on MN_LIS_, MN_LIS_, N_LIS_, and N_LIS_, Impregnated with GPL103 for the Different Growth Regimes Reported, Namely, Direct Condensation, Condensation–Coalescence, and Condensation–Coalescence–Shedding, of at Least Three Different Events^a^

		**condensation–coalescence**		
⟨*D*⟩ ∝ A*t*^μ^	**direct condensation**	**30–200 μm**	**200 μm - shedding**	**condensation–coalescence–shedding**
MN_LIS_GPL103_	(2.3 ± 0.1) *t*^(0.53±0.01)^	(0.25 ± 0.04) *t*^(1.02±0.04)^	(0.99 ± 0.28) *t*^(0.81±0.03)^	((1.0 ± 2.0)·10^–23^)*t*^(13.5±4.9)^
mN_LIS_GPL103_	(2.5 ± 0.1) *t*^(0.53±0.01)^	(0.15 ± 0.14) *t*^(1.26±0.08)^	(0.35 ± 0.15) *t*^(1.00±0.07)^	((1.8 ± 3.0) ·10^–24^)*t*^(16.0±4.9)^
N_LIS_GPL103_	(2.5 ± 0.1) *t*^(0.52±0.03)^	(0.16 ± 0.07) *t*^(1.13±0.07)^	(0.36 ± 0.10) *t*^(0.96±0.02)^	((1.8 ± 3.6) ·10^–14^)*t*^(17.2±15.1)^
n_LIS_GPL103_	(2.5 ± 0.1) *t*^(0.52±0.03)^	(0.28 ± 0.09) *t*^(1.04±0.10)^	(0.98 ± 0.55) *t*^(0.90±0.11)^	((1.7 ± 3.7) ·10^–33^)*t*^(14.4±3.0)^

a Note that we also divide the condensation–coalescence regime into microscopic for droplet sizes between 30 and 200 μm in diameter and macroscopic for droplet sizes between 200 μm and the droplet shedding size, as per the different observational technique adopted.

In the present case, for droplets with diameter sizes between 2 and 30 μm before coalescence, the relationship ⟨*D*⟩ ∝ A*t*^μ^ is independent of the surface structure, the infused lubricant, and/or the condensation time where all A and μ are found to be 2.5 ± 0.2 and 0.52 ± 0.02, respectively. See Supporting Information SI.9 and Table SI.VII and Table SI.VIII for more details on the calculated droplet growth values during the different condensation modes reported. We further note that despite the additional thermal resistance imposed by the micro-structures and the oil, no major differences on the droplet growth before coalescence are found when comparing the presence or absence of micro-structures and/or the type of lubricant; while up to 2 to 5 times less effective heat transfer through small sized droplets had been earlier theoretically reported as a consequence of the heat transfer resistance through the micro-structures.^51^

Once the droplets reach the transition radius *r*_*e*_ calculated as approximately 15 μm, in agreement with earlier works,^51^ droplets grow via direct condensation and coalescence. In this regime, we differentiate the analysis for droplets with sizes between 30 and 200 μm and between 200 μm and their shedding sizes. When looking at the smaller range of 30 to 200 μm, no major differences are found in the droplet growth and all the results can be self-contained within A = 0.21 ± 0.07 and μ = 1.11 ± 0.15. The power exponent coefficient greater than 1 is attributed to the stepwise droplet growth owed to the coalescence events with neighboring ones; in contrast to the linear increase on droplet size occurring during direct condensation. Note that the droplet growth coefficient reported here is larger than that reported by Beysens and Knobler where the radius of isolated droplets were found to grow proportional to *t*^0.75^ as a result of direct condensation and coalescence.^66^ As droplets grow bigger, for sizes between 200 μm and their shedding sizes, the heat transfer resistance through the droplet becomes more prominent and the overall droplet growth in the condensation-coalescence regime decreases. In such a regime the growth power exponent is slightly below or near 1; more specifically, the droplet growth in this regime follows A = 0.67 ± 0.31 and μ = 0.91 ± 0.10. To note is the greater standard deviation values for both coefficient A and droplet growth power exponent μ, which is attributed to the stochastic nature of the droplet size distribution surrounding the growing droplets analyzed.

While these first two regimes (direct condensation and condensation-coalescence) have been widely quantified and reported in the literature, the latter stages of condensation, that of condensation-coalescence-shedding has received lesser attention. Condensation-coalescence-shedding ensues as gravity forces overcome pinning forces, which in this work are predicted following eq 2, for droplets diameters ranging between 620 μm for mN_LIS_GPL103_ and 1160 μm for N_LIS_GPL107_. In this regime, the dynamics of droplet growth are governed mainly by the coalescence with neighboring droplets as the droplet sheds from the surface. Such droplet growth is here reported and quantified in detail for the first time. In this regime, the droplet growth is proportional to *t*^13.5^ to *t*^17.2^, which is at least 1 order of magnitude greater droplet growth power exponent μ than for the other condensations regimes reported. The occurrence of such exponentially increased droplet growth highlights the importance of enhancing shedding of small droplets and the droplet growth in the condensation-coalescence-shedding regime so to maximize heat transfer. In this regime, there is also a rather large variability in the droplet growth power exponent as well as on the constants, which are also attributed to the stochastic nature of the droplet size distribution surrounding the growing droplets analyzed.

We highlight here that despites the relatively high subcooling conditions d*T* = *T*_amb_ – *T*_sub_ = 25 °C, during condensation in humid air at atmospheric pressure, it is the diffusion of the water vapor through the noncondensable gases present in the environment limiting the condensation phenomenon, which leads to low droplet growth rates and condensation dynamics when compared to condensation under saturated steam conditions. It is expected that the different droplet growth exponents and proportionality constants reported in Table 6 will differ to account for the faster condensation dynamics taking place under saturated steam.^68,69^

### Heat Transfer

**Figure 6:**
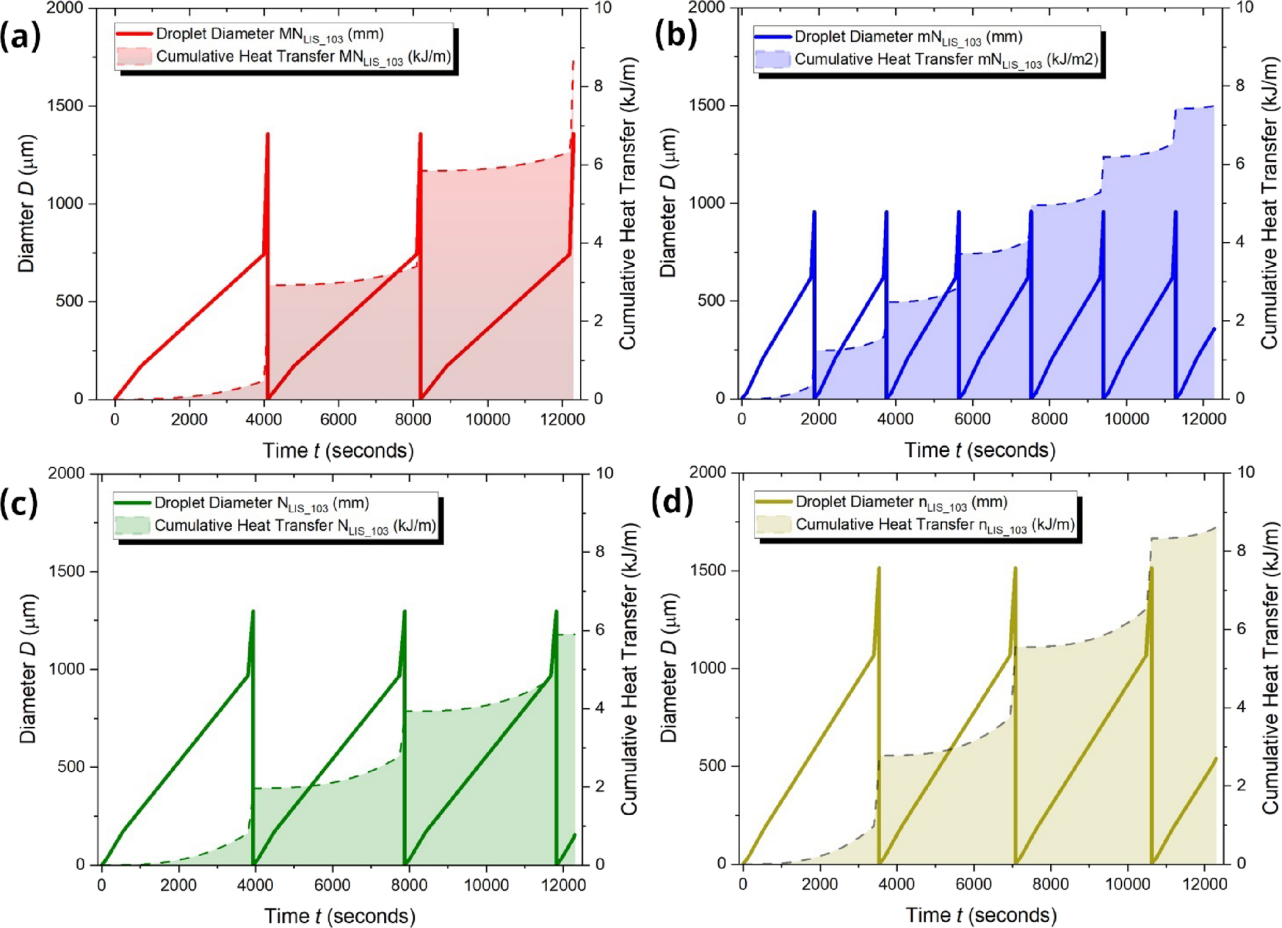
– Droplet diameter, *D* (μm), and cumulative heat transfer per unit length of shedding droplet (kJ/m) versus time, *t* (seconds) for (a) MN_LIS_103_, (b) mN_LIS_103_, (c) N_LIS_103_ and (d) n_LIS_103_. Droplet size is represented as a solid line while the cumulative heat transfer coefficient is represented as a shaded area delimited by a dashed line.

Although earlier works, making use of a coupled heat transfer resistance based model and the droplet size distribution, have reported a better heat transfer performance as the size of the structures decreases,^33,51^ the effect of the droplet shedding performance has not been accounted for. To this end, when accounting for the greater shedding performance and for the greater droplet growth during the here reported condensation-coalescence-shedding regime taking place on micro-structured LISs when compared to nano-structured LISs, earlier reported limitation owed to the additional heat transfer resistance imposed by the microstructures can be overcome. Next, Figure 6 presents the computed droplet size and cumulative heat transfer per unit of length for up to 12,500 s, which is equivalent to 3 to 6 droplet shedding cycles depending on the LIS surface studied. The droplet size or diameter was computed by making use of the different droplet growth rates regimes reported in Table 6 for direct condensation, condensation-coalescence and condensation-coalescence-shedding, while the cumulative heat transfer per unit length was calculated by making use of the droplet size/volume and the latent heat of condensation. See Supporting Information SI.10 Heat Transfer Considerations for more details on the computed droplet growth rates as well as cumulative heat transfer results.

Despites the 100% lower theoretical heat transfer performance reported on micro-/nano-structured LISs MN_LIS_ and mN_LIS_ when comparing to nano-structured N_LIS_ and n_LIS_,^51^ the better shedding performance of MN_LIS_ and mN_LIS_ as a consequence of the presence of micro-structures is able to achieve cumulative heat transfer per unit length values of the same order of magnitude as for N_LIS_ and n_LIS_, which are 8.77 kJ/m for MN_LIS_, 7.49 kJ/m for mN_LIS_, 5.90 kJ/m for N_LIS_ and 8.61 kJ/m for n_LIS_. The cumulative heat transfer coefficients reported in Figure 6 are further provided in a single figure in Figure SI.9 in the Supporting Information for compression. More specifically, the unexpected high cumulative heat transfer per unit length on micro-/nano-structured LIS (MN_LIS_ and mN_LIS_) able to overcome the additional heat transfer resistance imposed by the greater size of the structures, is owed to the faster occurrence of droplet growth and shedding during the condensation-coalescence-shedding regime. Moreover, the more frequent condensation-coalescence-shedding regime observed on micro-/nano-structured LIS, about all in the case of mN_LIS_, provides new refreshed area for droplet renucleation, growth, coalescence and shedding, which is characteristic of high heat transfer efficiency. Despite the greater condensate shedding performance reported on MN_LIS_ and mN_LIS_ and/or the lower heat transfer resistance earlier reported for N_LIS_ and n_LIS_, the similar cumulative heat transfer coefficients reported in Figure 6 are owed to the slow dynamics of condensation in turn limited by the diffusion of the water vapor toward the droplet interface in the presence of noncondensable gases.^68^ Nonetheless, in the presence of hierarchical MN_LIS_ and mN_LIS_, the empowered better droplet mobility shall benefit applications under isothermal conditions where the heat transfer across the surface does not play a role such as coatings or textiles.

Next, it is necessary to provide some insights on the stability of the infused oil. When looking into the different condensation experimental observations over the 4 h of duration, no appreciable differences on either the DWC performance or on the droplet growth rates reported in Table 6 are observed. The low standard deviation of the growth rates supports the rather uniform behavior over the entire duration of the experiments, which is further supported by the uniform droplet size densities on all four samples reported in the earlier work of Maeda *et al..*(51) In addition, long-term duration experiments on sample n_LIS_ over 11 h of condensation phase-change experimentation showed no degradation on the droplet shedding performance.^51^ Future considerations on the stability of the oil, which may deplete quicker from the regions in between the micro-structures^35,53,70^ in the case of MN_LIS_ and mN_LIS_, and/or be carried out by the cloaked droplets, must be taken into account and deserves the attention of the scientific community.

While current strategies focus on minimizing the heat transfer resistance between the condensing surface and the droplets, the current investigation highlights the paramount importance of efficient shedding and the condensation-coalescence-shedding regime by the implementation of micro-structures, which effectively decrease the interactions between the droplet and the surface and eventually increases the heat transfer. This is a promising strategy toward the design and optimization of advanced engineered surfaces for fluid manipulation and thermal management applications.

## Conclusions

The greater slippery nature of hierarchical micro-/nano-structured liquid-infused surfaces (LISs) when compared to solely nano-structured ones is here demonstrated both by experimental observations and by a force balance analysis. The greater velocities and the smaller size of the shedding droplets evidence the greater mobility and shedding performance during dynamic condensation of droplets on hierarchical micro-/nano-structured LISs. In addition, direct optical microscopy observations through condensing droplets are able to resolve the different intimate interactions between droplets and the various LIS structures at the at the droplet–LIS interface supporting the different condensation behaviors reported. A revisited tangential to the surface force balance accounting for the decrease in the effective pinned fraction of the contact line upon the inclusion of micro-structures on our LISs remarkably agrees with our experimental observations. Moreover, the enhanced refreshing frequency of smaller droplets can enhance the heat transfer performance on hierarchical micro-/nano-structured LISs, which can overcome earlier heat transfer limitations suggested by the heat transfer resistance imposed by the surface/structures. Methodology and findings reported here are of great importance for the effective design of surfaces with enhanced heat transfer performance and for applications where droplet mobility is paramount such as anti-icing, self-cleaning, antifogging, fluid manipulation, and thermal management applications, among others.

## Materials and Methods

### Surface Fabrication

Two types of hierarchical micro- and nano-structured superhydrophobic surfaces (SHSs) varying in size and density of the microstructures and two nanostructured ones were fabricated as in the work of Zhang *et al..*(50,54) Pristine copper plates of 10 × 10 mm^2^ and thickness of 500 μm were cleaned in an ultrasonic bath in sequence using acetone, ethanol and distilled water prior to drying with nitrogen gas to remove contaminants. Thereafter, surfaces were immersed in a solution of 10 wt % of HCl–H_2_O to remove the oxide layer from the copper surface, and then samples were further cleaned in an ultrasonic bath with distilled water followed by drying with nitrogen. Next, to create the different size and density of the microstructures on hierarchical micro-/nano-structured substrates, two of the samples were subjected to facile and easily scalable etching in a solution of 0.48 wt % H_2_O_2_–H_2_O and 1.89 mol/L HCl–H_2_O, as in refs (50, 47), whereas the other two solely nano-structured samples were not subjected to additional etching. Bigger size and greater density of micro-structures were conferred by dipping the copper plates for longer time at higher temperature (1 h at 60 °C versus 20 min at 17 °C).^50,54^ The different size and density of the micro-structures were further confirmed by scanning electron microscopy SEM and 3D laser optical microscopy included in Figure SI.1a–h in the Supporting Information. To confer the surfaces with the necessary nano-scale roughness for the effective infusion and stability of the lubricant, etched substrates and cleaned pristine ones were further oxidized in an aqueous solution of 2.5 mol/L NaOH–H_2_O and 0.1 mol/L ((NH)_4_S_2_O_8_–H_2_O) for 30 min at 70 °C for MN_LIS_, mN_LIS_ and N_LIS_.^48,50^ The last of the nonetched samples, on the other hand, was further oxidized in the same aqueous solution of 2.5 mol/L NaOH–H_2_O and 0.1 mol/L ((NH)_4_S_2_O_8_–H_2_O) for 50 min at 15 °C for n_LIS_. For simplicity, we will refer to the hierarchical micro- and nano-structured samples as MN_LIS_ for high density and big size of micro-structures and mN_LIS_ for small size and low density of micro-structures. On the other hand, solely nano-structured ones are referred as N_LIS_ and n_LIS_. After surface oxidation, all samples were rinsed with deionized water and baked at 180 °C for a further hour to completely remove any presence of water. Then, baked samples were immersed in 1% POTS-ethanol for 12 h at *T*_amb_, which rendered them hydrophobic. The hydrophobicity of the nanostructures is a necessary condition for liquid-infused surfaces (LISs) in order to induce the more wetting affinity of the lubricant infused within the substrate micro- and/or nano-structures when compared to water.^54^ All chemicals were purchased from Sinopharm Chemical Reagent Co., Ltd. (China). After etching, oxidation, and hydrophobization of the surfaces, each set of the four samples (MN_LIS_, mN_LIS_, N_LIS_, and n_LIS_) was immersed into Krytox general-purpose lubricant 103 (GPL103) from DuPont (USA), while a different set of the same four samples was immersed into Krytox general-purpose lubricant 107 (GPL107) also from DuPont (USA), henceforth referred to as GPL103 and GPL107. After immersion in the lubricant, the LISs were slowly removed and placed vertically for an hour in order to remove any excess of lubricant prior to observations.

### Surface Characterization

Scanning electron microscopy (SEM) and 3D laser optical microscopy profiles of the superhydrophobic MN_LIS_, mN_LIS_, N_LIS_, and n_LIS_ before lubricant impregnation are presented in Figure 1g–j and in Figure SI.1a–h. SEM was carried out in a 3D Versa dual beam environmental scanning electron microscope from FEI Company (Hillsboro, Oregon, USA), whereas 3D laser optical microscopy was carried out in an LEXT OLS4000 from Olympus (Japan).

### Lubricant Characterization

Krytox General-Purpose Lubricant 103 (GPL103) from DuPont (USA) with a density of 1.88 kg/dm^3^ and a kinematic viscosity of 82 centistokes at 20 °C, and a Krytox general-purpose lubricant 107 (GPL107) also from DuPont (USA) with a density of 1.92 kg/dm^3^ and a viscosity of 1535 centistokes at 20 °C, were utilized. The surface tension of the Krytox GPL103 and GPL107 in both air and water was performed in a custom-built pendant droplet setup and further analyzed using ImageJ: Pendent_Drop: an ImageJ plugin to measure the surface tension from an image of a pendant drop developed by Daerr and Mogne.^55^ On one hand, in the case of GPL103 the lubricant surface tension in air *γ*_oa_ and that of the lubricant in water *γ*_ol_ were measured as 16.1 ± 0.5 and 53.0 ± 2.0 mN/m, respectively, which are in close agreement with values reported in the literature.^40,56^ The spreading coefficient of lubricant in water *S*_ow_ equals 3.64. Since the spreading coefficient is greater than 0, the lubricant may cloak the condensing droplets. In addition, the thickness of the cloaking film for Krytox GPL 103 can be estimated as , where *A*_*H*_ is the Hamaker constant (*A*_*H*_ = 10^–18^ J) and *R* is the droplet radius.^40,54^ Then, for a 3 μL, i.e., *R* = 0.9 mm below the capillary length for water, the thickness of the cloaking film is estimated as *δ*_lubricant_ = 73 nm.^54^ On the other hand, in the case of GPL107 the lubricant surface tension in air *γ*_oa_ and that of the lubricant in water *γ*_ol_ were measured as 17.4 ± 0.5 and 54. ± 2.0 mN/m, respectively, also in agreement with values reported in the literature.^40,56^ The spreading coefficient of lubricant in water *S*_ow_ equals 1.11 and the thickness of the cloaking film is estimated as *δ*_lubricant_ = 72 nm.^54^

### Condensation Experimental Observations

Experimental observations were carried out in PR-3KT environmental chamber from ESPEC Corp. (Japan) at *T*_amb_ = 30 °C ± 1 °C and RH = 90% ± 5%. A vertical Peltier stage is connected to a PID controller and to a cooling bath. A custom-built copper block of the same size as the LISs (10 × 10 mm^2^) is inserted in a thermally insulating TEFLON block placed on the Peltier stage to ensure one-dimensional heat transfer between the Peltier stage and the LISs. The LIS was attached to the Cu block using a double side carbon tape. A thermocouple is also set at the center of the copper block few millimeters below the LIS. The temperature on the LIS was found within ±1.5 °C when compared to *T*_sub_ displayed by the PID controller. Before experimental observations, to ensure homogeneous conditions within the chamber *T*_amb_ and RH were kept constant for 30 min. To avoid condensation prior to experimental observations, *T*_sub_ was kept above the dew point at 35 °C. Thereafter, experimental observations were carried out at *T*_sub_ = 5 °C for 4 h where up to 14 droplets shed off events took place on MN_LIS_, N_LIS_, and n_LIS_ while up to 28 droplets shed off events occurred on mN_LIS_ as a consequence of the better droplet shedding behavior reported for this LIS in this work. The experimental environmental conditions of *T*_amb_ = 30 °C ± 1 °C and RH = 90% ± 5% were chosen. A 1.4 Megapixels CCD camera Sentech STC-MC152USB with a RICOH lens with 30 mm spacing and a LED illuminating from above was used for macroscopic experimental observations. Experiments were recorded at a frame rate of 5 fps for a period of 4 h, while videos were thereafter reduced for a 0.5 fps for analysis; Supporting Information videos include macroscopic observations at 1 frame per minute reproduced at 1 fps. Meanwhile, a high-resolution zoom lens Keyence VH-Z50L (Japan) at 500× magnification providing a field of view of 605 × 457 μm^2^ coupled to a 1.4 Megapixels CCD camear Sentech STC-MC152USB for a field of view of 605 × 457 μm^2^ was used for experimental observations of droplet growth with sizes in the order of tens to hundreds of μm. Picture and schematic of the two different condensation experimental setups can be found in Figure SI.6 and Figure SI.7 in the Supporting Information.

## Supporting Information Available

Additional detailed information on surface topography characterization (SI.1), microstructure solid fraction estimation (SI.2), surface-LIS characterization (SI.3), wettability and contact angle characterization (SI.4), lubricant surface tension characterization (SI.5), condensation experimental observations (SI.6), data extraction and analysis (SI.7), force balance analysis (SI.8), droplet growth (SI.9), and heat transfer considerations (SI.10) (PDF)
Movie showing the condensation behavior over 4 h for 1 frame per minute reproduced at 1 fps and reduced frame size to 640 × 480 px on MN_LIS_ (AVI)
Movie showing the condensation behavior over 4 h for 1 frame per minute reproduced at 1 fps and reduced frame size to 640 × 480 px on mN_LIS_ (AVI)
Movie showing the condensation behavior over 4 h for 1 frame per minute reproduced at 1 fps and reduced frame size to 640 × 480 px on N_LIS_ (AVI)
Movie showing the condensation behavior over 4 h for 1 frame per minute reproduced at 1 fps and reduced frame size to 640 × 480 px on n_LIS_ (AVI)

## Supplementary Material

am3c14232_si_001.pdf

am3c14232_si_002.avi

am3c14232_si_003.avi

am3c14232_si_004.avi

am3c14232_si_005.avi
