# Supplementary material for: Nanorough Is Not Slippery Enough: Implications on Shedding and Heat Transfer
Source: ACS Appl Mater Interfaces. 2024 Jan 2;16(1):1779–93. doi: 10.1021/acsami.3c14232 (PMC10788867; doi:10.1021/acsami.3c14232)
Supplement: Supplementary file 1 — am3c14232_si_001.pdf [file am3c14232_si_001.pdf]

Supporting Information - Nano-rough is not Slippery Enough: Implications on Shedding and Heat Transfer

Daniel Orejon[1,2,*], Yota Maeda[3], Peng Zhang[4], Fengyong Lv[5], Yasuyuki Takata[1,2]

[1]Institute for Multiscale Thermofluids, School of Engineering, University of Edinburgh, EH9 3BF, Scotland, UK

[2]International Institute for Carbon-Neutral Energy Research (WPI-I2CNER), Kyushu University, 744 Motooka, Nishi-ku, Fukuoka 819-0395, Japan

[3]Department of Mechanical Engineering, Thermofluid Physics Laboratory, Kyushu University, 744 Motooka, Nishi-ku, Fukuoka 819-0395, Japan

[4]Institute of Refrigeration and Cryogenics, Shanghai Jiao Tong University, Shanghai 200240, China

[5]School of Urban Construction and Safety Engineering, Shanghai Institute of Technology, Shanghai, 201418 China

ABSTRACT

Lowering droplet-surface adhesion via the effective implementation of Lubricant Infused Surfaces (LISs) has received important attention in the past years as owed to its intrinsic relevance to industrial and everyday applications. LISs offer enhanced droplet mobility, low sliding angles and the recently reported slippery Wenzel state, amongst others, empowered by the presence of the lubricant infused in between the structures, which eventually minimises the direct interactions between liquid droplets and LISs. Current strategies to increase heat transfer during condensation phase-change relay on minimizing the thickness of the coating as well as enhancing condensate shedding. While further surface structuring may impose an additional heat transfer resistance, the presence of microstructures eventually reduces the effective condensate–surface intimate interactions with the consequently decreased adhesion and enhanced shedding performance, which is investigated in this work. This is demonstrated by macroscopic and optical microscopy condensation experimental observations paying special attention at the liquid–lubricant and liquid–solid binary interactions at the droplet–LIS interface, which is further supported by a revisited force balance at the droplet triple contact line. Moreover, the occurrence of a condensation–coalescence–shedding regime is quantified for the first time with droplet growth rates one and two orders of magnitude greater than during condensation–coalescence and direct condensation regimes, respectively. Findings presented here are of great importance for the effective design and implementation of LISs via surface structure endowing accurate droplet mobility and control for applications such as anti-icing, self-cleaning, water harvesting, and/or liquid repellent surfaces as well as for condensation heat transfer.

* Author to whom correspondence should be addressed: Daniel Orejon, d.orejon@ed.ac.uk

SI.1 - SURFACE CHARACTERIZATION

Scanning Electron Microscopy (SEM) and 3D laser optical microscopy profiles of the superhydrophobic MN_{LIS}, mN_{LIS}, N_{LIS} and n_{LIS} before oil impregnation are presented in Figure SI.1. SEM was carried out in a 3D Versa Dual Beam Environmental Scanning Electron Microscope from FEI Company (Hillsboro, Oregon, USA), whereas 3D laser optical microscopy was carried out in a LEXT OLS4000 from Olympus (Japan).

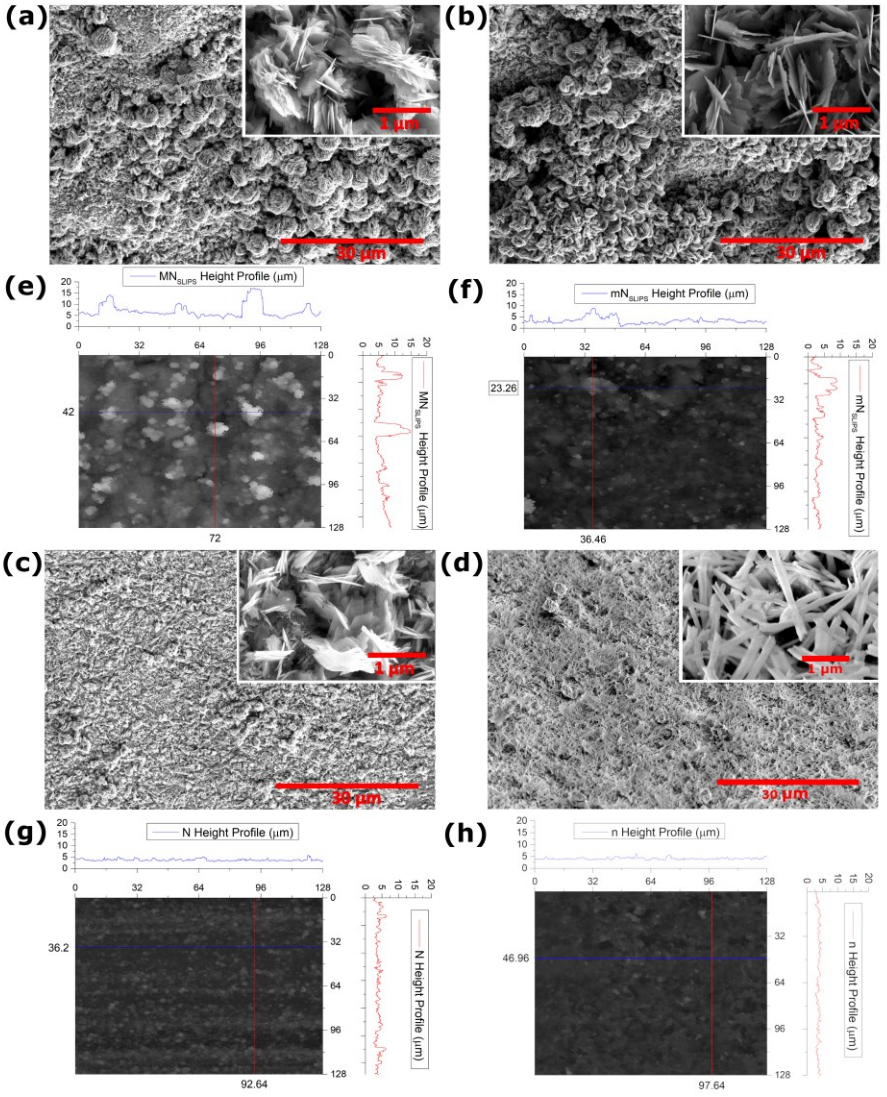

Figure SI.1 – SEM of (a) MN_{LIS}, (b) mN_{LIS}, (c) N_{LIS} and (d) n_{LIS} prior to impregnation. Scale bar is 30 µm. Insets include high magnification SEM of the nanostructures with scale bar of 1 µm. 3D laser optical microscopy and 2D profiles for (e) MN_{LIS}, (f) mN_{LIS}, (g) N_{LIS} and (h) n_{LIS} prior to impregnation. Field of view of the 3D profiles is 128 x 128 µm^2 with a maximum height profile of 20 µm. Greyscale intensity of the 2D surface profile. SEM magnification figure (a) to (d) insights are from Reference SI-1.[1]

From SEM and the 3D laser optical microscopy profiles, the greater size and density of hierarchical MN_{LIS} sample when compared to hierarchical mN_{LIS} is evident. In addition, the absence of microstructures on N_{LIS} and on n_{LIS} is also highlighted. Average surface roughness S_{RMS} was extracted from 3D laser optical microscopy observations.

SI.2 - SOLID FRACTION OF THE MICRO-STRUCTURES

From SEM and 3D laser optical microscopy profiles we estimate the solid-fraction of micro-structures, *Ω*. 2D laser optical microscopy profile above certain microstructure height threshold is plotted with Origin. Height thresholds were chosen as half of the maximum distance between peak and valley S_z reported in Table SI.2 as 8 µm and 5 µm on MN_{LIS} and mN_{LIS}, respectively. Then, the surface area of the microstructures with height above chosen threshold is extracted by ImageJ. 3D laser optical microscopy profile and ImageJ analysis for MN_{LIS} for a threshold height of 8 µm and for mN_{LIS} for a height threshold 5 µm are presented in Figure SI.2 and Figure SI.3, respectively.

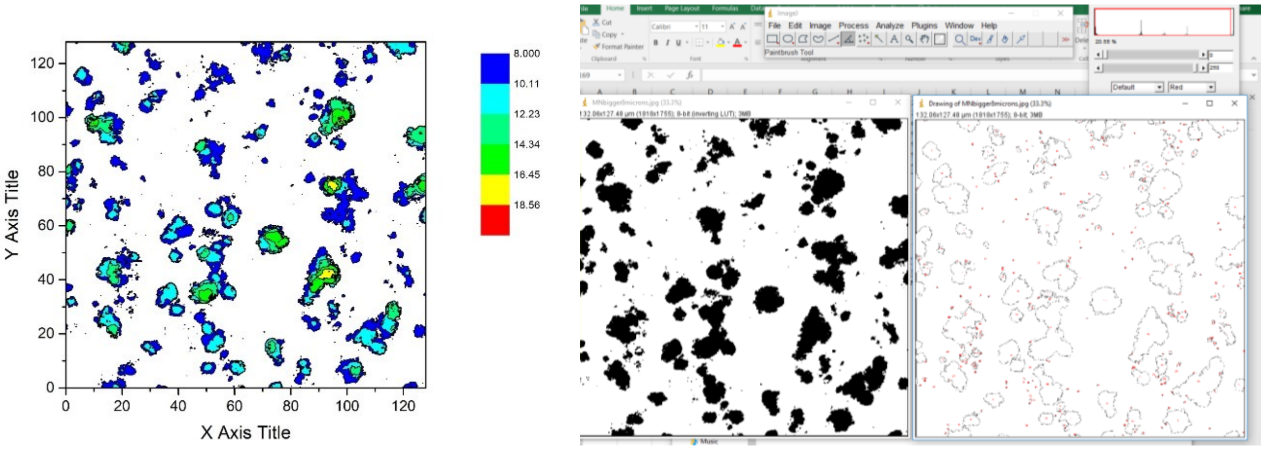

Figure SI.2 - 3D laser optical microscopy profile for a threshold height of 8 µm and ImageJ analysis for MN_{LIS} yielding a solid fraction of micro-structures of *Ω*=30.5%.

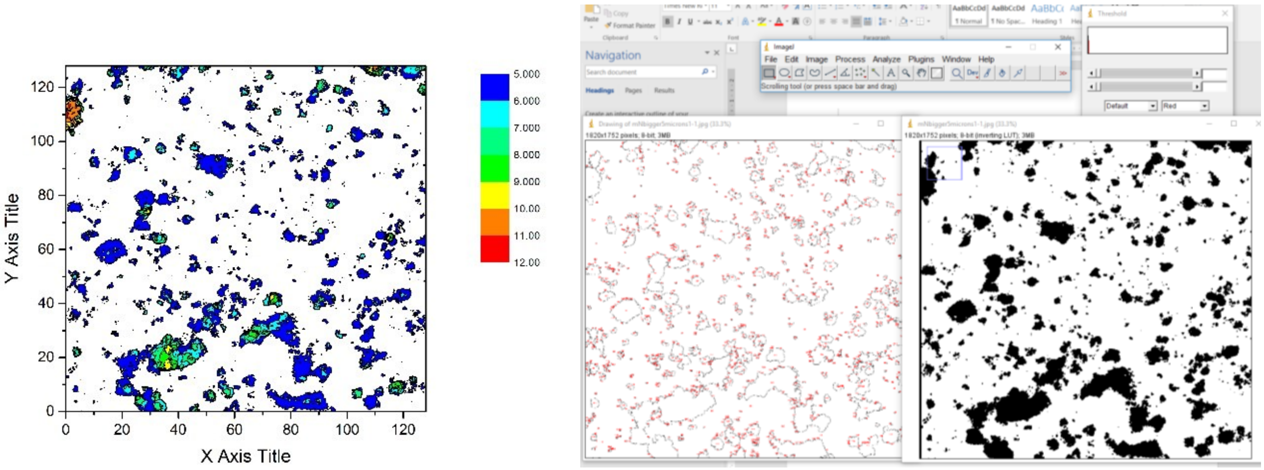

Figure SI.3 - 3D laser optical microscopy profile for a threshold height of 5 µm and ImageJ analysis for mN_{LIS} yielding a solid fraction of micro-structures of *Ω*=19.6%.

SI.3 - SURFACE-LUBRICANT CHARACTERIZATION

Further characterisation of the wetting properties of the oil and the superhydrophobic surfaces aiming to provide the full wetting picture of the quaternary system surface-lubricant-condensate-air is introduced next. The equilibrium contact angle of the oil on the micro- and the nano-structured superhydrophobic surfaces is measured. Typically, for the complete wetting and encapsulation of the structures by the lubricant, the measured equilibrium contact angle of the lubricant on the different structured surfaces should be $\theta_{os(a)} \sim 0°$ and hence the lubricant encapsulates such structures.[2] For a finite contact angle between the lubricant and the SHS below the critical contact angle for hemiwicking $\theta_{os(a)} < \theta_c$, the impregnated-emerged state ensues where the lubricant spreads through the micro- and nano-structures while the top of the nano-structures are exposed to the ambient.[3, 4] For a contact angle above the critical angle $\theta_{os(a)} > \theta_c$, the dry state occurs.[2]

The critical angle for hemiwicking θ_c is estimated as $\theta_c = \cos^{-1}(1-f)/(\varphi - f)$, where f is the solid fraction included in Table SI.I and φ as the roughness factor due to the presence of micro-structures.[3, 4] The solid fraction f, on the other hand, is estimated from the advancing contact angle on the superhydrophobic surface prior to oil impregnation as: $f = (\cos\theta_a + 1)/(\cos\theta_a^{\text{flat}} + 1)$.[5, 6] Whereas $\varphi = 1 + (4ah/(a+b)^2)$ where a is the characteristic microstructure length extracted from the 2D profile by assuming the micro-structures as circular micro-pillars. The surface area of the micro-structures A is measured from Figure SI.2 and SI.3 and then the characteristic length is approximated as $a = (4A/\pi)^{0.5}$ and h is extracted from the height profiles included in Figure SI.1. $(a+b)^2$ is the area of the 2D laser optical microscopy observations included in Figure SI.I equals 128^2 μm^2. For the studied surfaces MN_{LIS} and N_{LIS} characterisation values are included in Table SI.I.

Table SI.I – Roughness factor φ, solid fraction f, and critical angle for hemiwicking θ_c for MN_{LIS} and N_{LIS} are included:

	φ	f [6]	θ_c [3, 4]
**MN_{LIS}**	1.347	0.128	44° ± 2°
**N_{LIS}**	1	0.115	32° ± 3°

Next, the equilibrium contact angle of the lubricant on the different LISs before impregnation upon lubricant sessile droplet deposition are measured in custom-built goniometer and analysed using ImageJ and a snake-based approach plugging.[7, 8] The characteristic finite contact angle $\theta_{os(a)}$ for GPL103 on MN_{LIS} and N_{LIS} before impregnation was found to be independent of the surface structure underneath the oil and equals 8° ± 3°. Whereas in the case of GPL107, the equilibrium contact angle on the micro-structured MN SHS was $\theta_{os(a)} \sim 18° \pm 3°$ and on the nanostructured N SHS $\theta_{os(a)} \sim 11° \pm 3°$. The finite contact angles reported between the lubricant and the structured surfaces below the calculated critical contact angles for hemiwicking included in Table SI.I confirm that the ternary system solid-oil-air shall behave in the impregnated-emerged state.

SI.4 - CONTACT ANGLE CHARACTERIZATION OF LISs

In addition, advancing and receding contact angles for water, θ_a and θ_r on the different LISs surfaces for both GPL103 and GPL107 oils were measured in custom-built goniometer and analysed using ImageJ and a snake-based approach plugging.[7, 8] Standard deviation was calculated from at least 5 independent measurements. Table SI.II summarizes the data on the characterization of SHSs before impregnation, *i.e.*, average surface roughness S_{RMS}, nano-structure solid fraction f, micro-structure solid fraction, along with the advancing and receding contact angles θ_a and θ_r and the contact angle hysteresis *CAH* on the different structured surfaces after impregnation with GPL103 and GPL107. We note here that on MN_{LIS} and N_{LIS} f was assume to be that of the nano-structured LIS NLIS, while ϕ on MN_{LIS} and N_{LIS} was estimated as reported in SI.3 above and on NLIS and nLIS was assumed as 0 as per the absence of nano-structures demonstrated via SEM and 2D laser optical microscopy in Figure SI.1.

Table SI.II – Substrate structural characterization of MN_{LIS}, mN_{LIS}, N_{LIS} and n_{LIS} as: surface roughness S_{RMS} (µm), maximum distance between peak and valley S_z (µm), nano-structures solid fraction f (-), micro-structures solid fraction ϕ (-), and Contact Angle Hysteresis ($CAH_{\_SHS}$) prior to impregnation. And surface wettability characterisation as: advancing contact angle θ_a (deg), receding contact angle θ_r (deg) and contact angle hysteresis *CAH* (deg) of MN_{LIS}, mN_{LIS}, N_{LIS} and n_{LIS} impregnated with GPL103 and GPL107. Each of the wettability measurements reported include the average and standard deviation for at least 5 different independent measurements.

	MN_{LIS}	mN_{LIS}	N_{LIS}	n_{LIS}
S_{RMS} (µm)	2.1	1.4	0.8	0.5
S_z (µm)	16	10	-	-
f	0.11	0.11	0.11	0.10
ϕ	0.31	0.20	-	-
$CAH_{\_SHS}$	< 1°	< 1°	< 1°	< 1°
θ_{a_GPL103} (deg)	114° ± 2°	118° ± 2°	117° ± 2°	118° ± 2°
θ_{r_GPL103} (deg)	111° ± 4°	115° ±4°	114° ± 2°	115° ± 3°
CAH_{GPL103}	3° ± 2°	3° ± 2°	3° ± 3°	3° ± 3°
θ_{a_GPL107} (deg)	113° ± 2°	114° ± 3°	114° ± 2°	114° ± 3°
θ_{r_GPL107} (deg)	110° ± 2°	110° ± 2°	110° ± 2°	111° ± 3°
CAH_{GPL107}	3° ± 2°	4° ± 3°	4° ± 2°	3° ± 3°

SI.5 - LUBRICANT SURFACE TENSION CHARACTERIZATION

A custom-built surface tension experimental apparatus to measure the surface tension of the lubricant both in air γ_{oa} and in water γ_{ol} was used. Schematics of the custom-built experimental apparatus is included in Figure SI.4. Thereafter snapshots of the lubricant pending drops before detachment were analysed by an ImageJ plugin developed by Daerr and Moogne.[9]

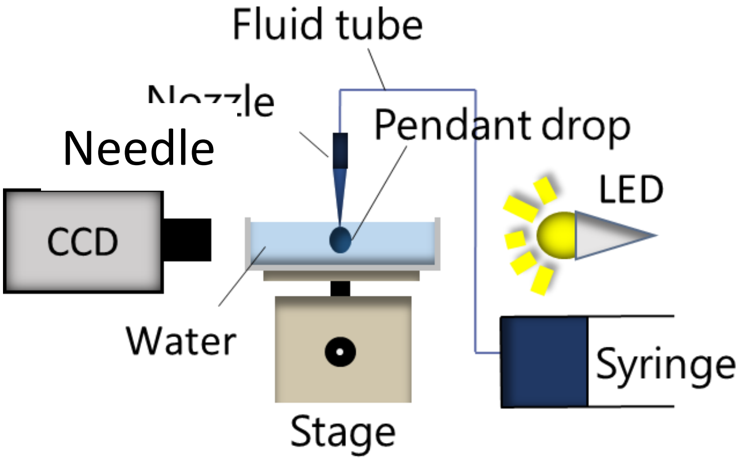

Figure SI.4 – Schematics of the experimental apparatus used to measure the lubricant surface tension in water. Experimental apparatus comprises: dosing system with a needle and a syringe, a z-axis stage, a CCD camera and a LED light to allow for the necessary contrast. In the case of the surface tension lubricant-air, measurements were carried out in ambient, whereas in the case of lubricant-water surface tension, a cube cuvette with dimensions greater than the size of the droplet was used.

The recording was then started and a sessile pendant droplet was slowly growth at the tip of the needle. Then the last frame before the lubricant droplet detachment was processed with Image J. Figure SI.5 presents characteristic snapshot in ImageJ used for the analysis of the surface tension:

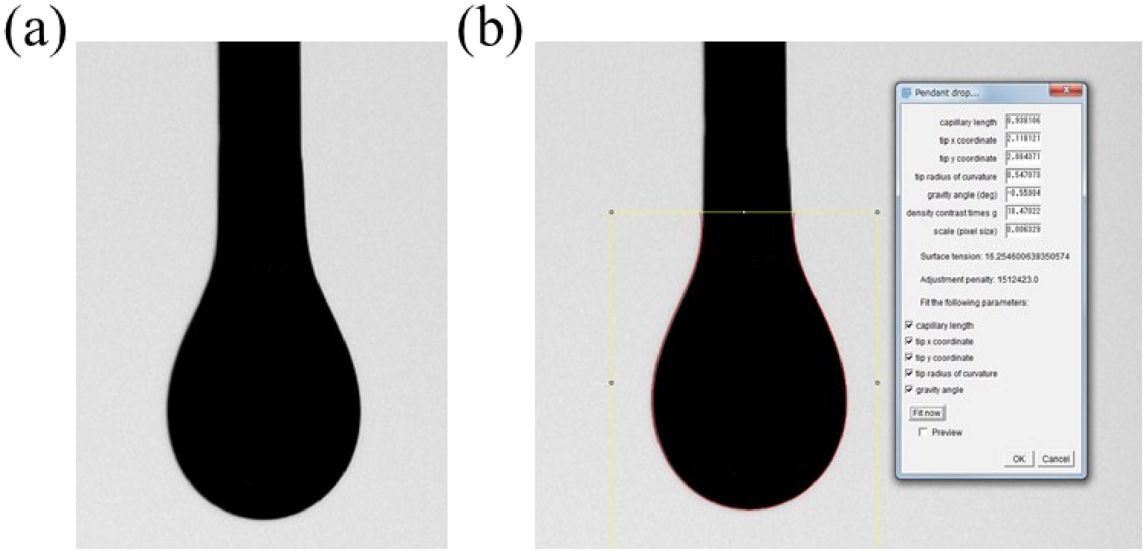

Figure SI.5 – Characteristic snapshots of (a) a GPL103 lubricant pendant droplet being produced at the tip of a needle after image processing to enhance the contrast between the pendant droplet and air ambient and (b) the same pendant droplet being analysed after automatic optimization of the parameters using the ImageJ plugin developed by Daerr and Moogne.[9]

SI.6 - CONDENSATION EXPERIMENTAL OBSERVATIONS

Picture and schematic of the experimental setup for macroscopic observations is included in Fig. SI.6:

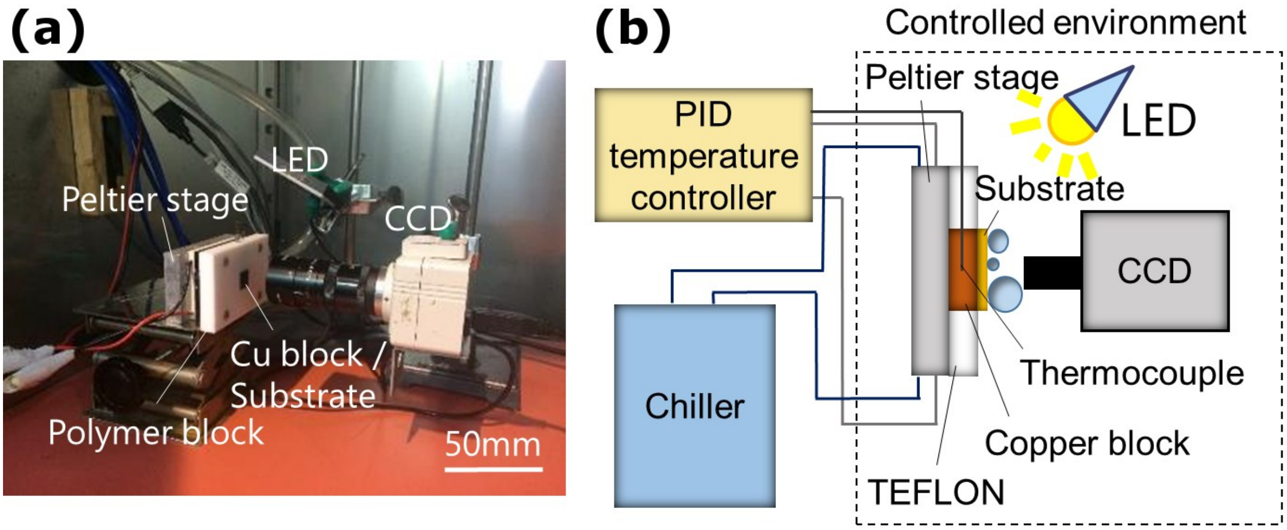

Figure SI.6 – (a) Picture of the experimental setup including Peltier stage, chiller and thermocouple connected to a PID controller, Cu-TEFLON block and LIS sample, LED light, CCD camera with RICOH lens and 30 mm spacing for experiments at the macroscale. (b) Schematic of the complete experimental setup including environmental chamber PID controller and chiller.

Picture and schematic of the Optical Microscopy Setup including optical microscopy lens Keyence VH-Z50L (Japan) and CCD camera used in this investigation as in the work of Maeda et al.[1] can be found below in Figure SI.7:

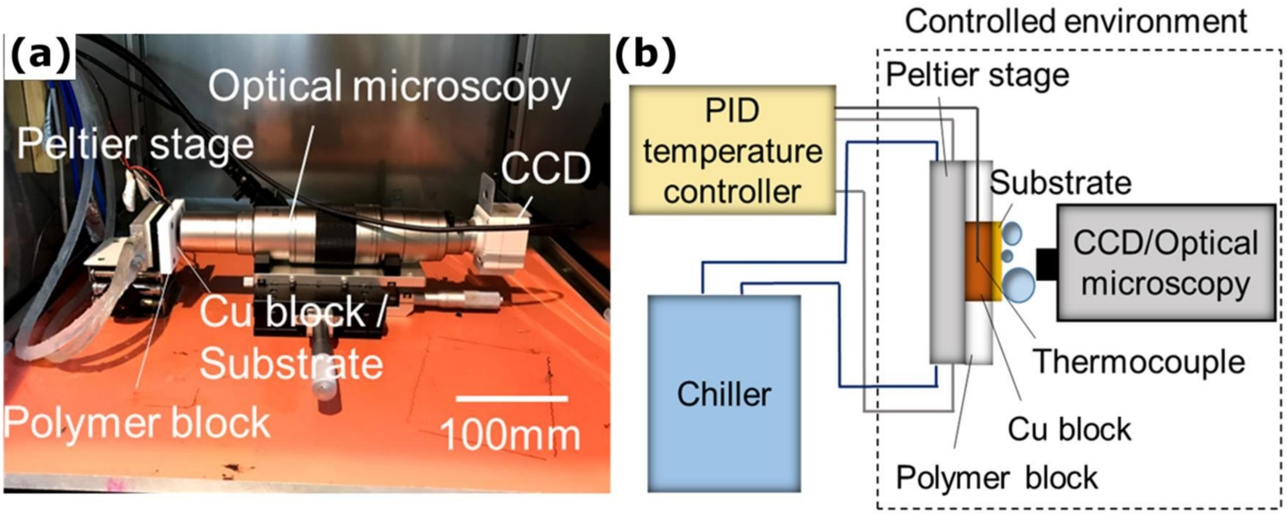

Figure SI.7 – (a) Picture of the experimental setup including Peltier stage, chiller and thermocouple connected to a PID controller, Cu-TEFLON block and LIS sample, LED light, CCD camera with a Keyence VH-Z50L lens and 30 mm spacing for experiments at the macroscale. (b) Schematic of the complete experimental setup including environmental chamber PID controller and chiller.

SI.7 - DATA EXTRACTION AND ANALYSIS

Droplet shedding data analysis on all four LISs is described next. From macroscopic observations, we track the position and the size of mobile droplets in time using ImageJ.[7] Circle shapes are manually fitted and the droplet centroid position (x , y) and its area (A) are extracted. From the droplet area we calculate the droplet curvature radius as: $R = \sqrt{A/\pi}$ and from the droplet position we calculate the velocity of the droplet, v, as: $v = \sqrt{(y_0 - y_1)^2 + (x_1 - x_0)^2}/t$ where (x_0 , y_0) and (x_1 , y_1) are the droplet centroid position between two frames with t as the time between frames. Then droplet velocities v (µm/s) versus droplet radius R (mm) on MN_{LIS}, mN_{LIS}, N_{LIS} and n_{LIS} impregnated with GPL103 and GPL107 are presented in Figure SI.7 and Figure SI.8, respectively (Figure 3 in the main manuscript). Droplet velocities where the change in droplet radius between frames due to condensation and/or coalescence is greater than 1% have been disregarded from the data analysis reported in both Figure SI.7 and Figure SI.8. Velocities reported in Figure SI.7 and Figure SI.8 are for the different mobility events of at least 2 or 3 different droplets shedding from the different LISs. Note that over the 4 hours duration of the experiments up to 14 droplets shed off the surface; however, only 3 droplets were fully analysed as other droplets may have been partially outside the field of view or undergone major coalescence events.

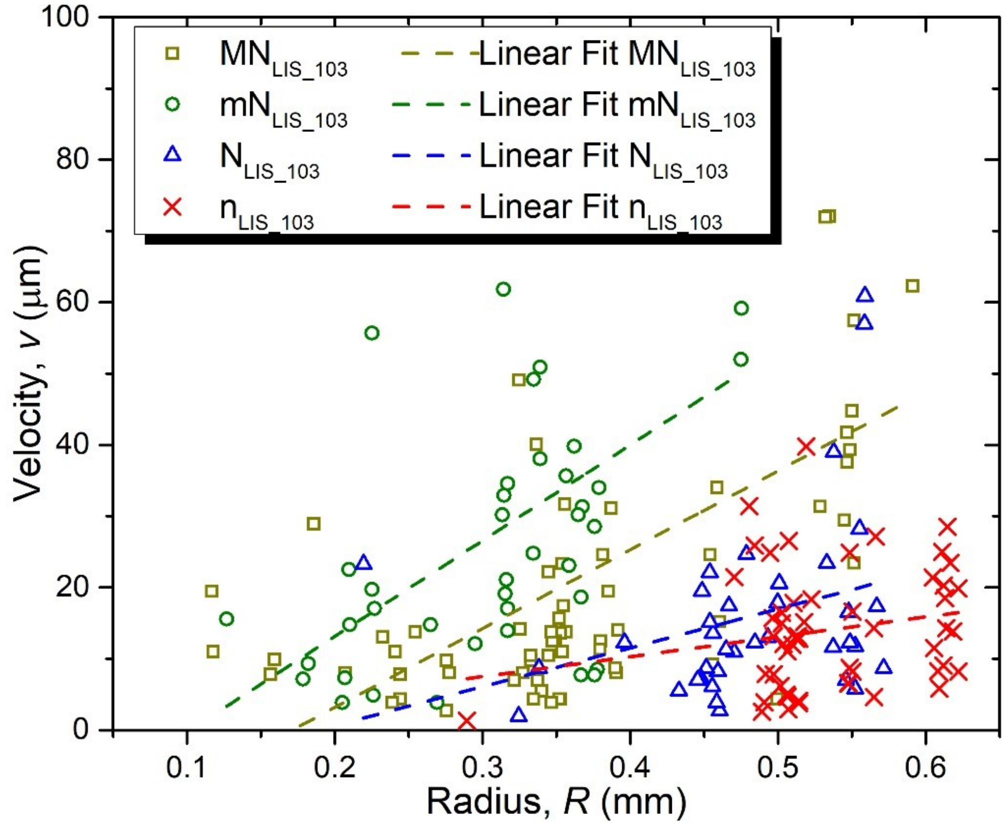

Figure SI.7 – Droplet velocity, v (µm/s), represented *versus* droplet radius, R (mm), on (dark yellow squares) MN_{LIS}, (green circles) mN_{LIS}, (blue up-triangles) N_{LIS} and (red crosses) nLIS, with GPL103 as the lubricant oil. Linear trend is included for comparison.

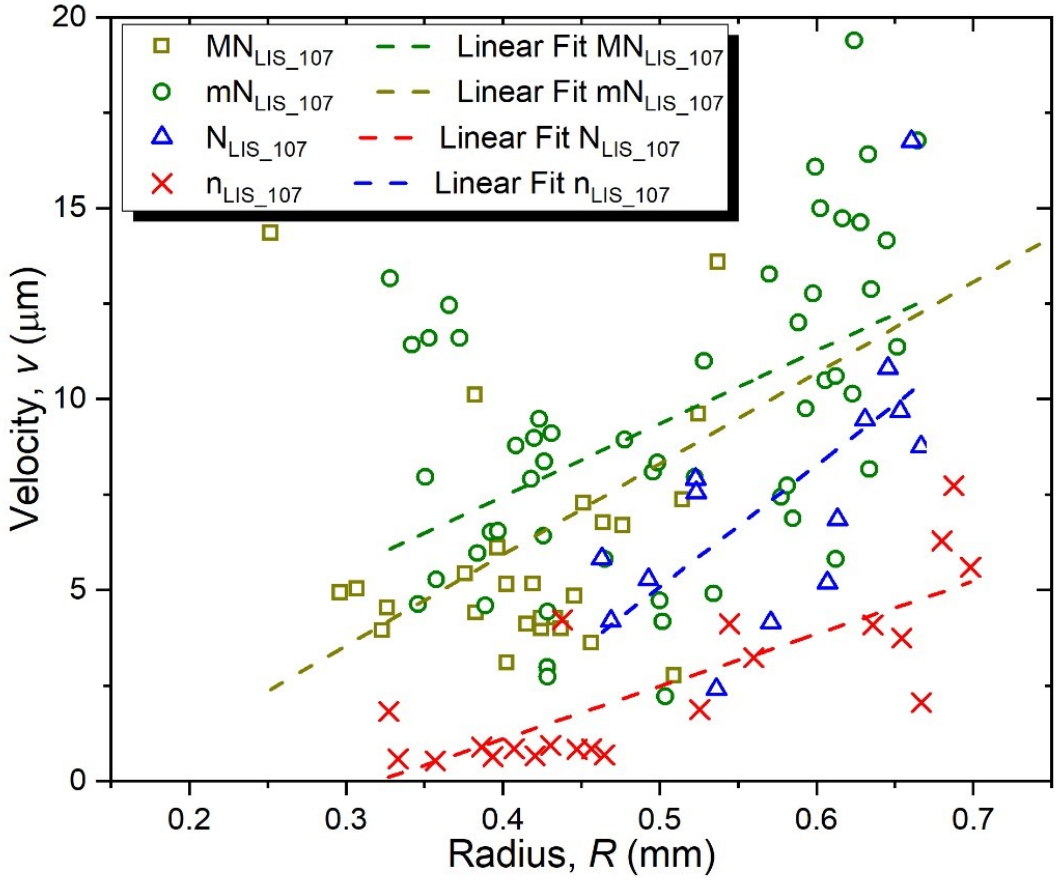

Figure SI.8 – Droplet velocity, *v* (µm/s), represented *versus* droplet radius, *R* (mm), on (dark yellow squares) MN_{LIS}, (green circles) mN_{LIS}, (blue up-triangles) N_{LIS} and (red crosses) n_{LIS}, with GPL107 as the lubricant oil. Linear trend is included for comparison.

SI.8 – FORCE BALANCE ANALYSIS

When a droplet sits on an inclined surface, there is typically a pinning force keeping the droplet attached to the surface, *i.e.*, pinning force F_{pin}, and a force pulling the droplet down due to gravity effects, *i.e.*, gravitational depinning force F_g. For the droplet to move, F_g must overcome F_{pin} as in Equation SI.1:[10-15]

$$F_g - F_{\text{pin}} = \rho V g \sin\alpha - \gamma_{lg}\pi D_b(\cos\theta_r - \cos\theta_a) > 0 \qquad \text{Eq. SI.1}$$

Where V is the droplet volume, g is gravity, ρ is the density of water, α is the inclination of the surface and equals 90° hence sinα equals 1, D_b is the droplet wetting base diameter equals $D \sin\theta_a$, and γ_{lg} is the liquid-vapour surface tension. From Equation SI.1, F_g function of the droplet weight and the surface inclination angle and F_{pin} function of the droplet base wetting perimenter πD_b and of the contact angle hysteresis: CAH = $\theta_a - \theta_r$ are calculated and presented for MN_{LIS}, mN_{LIS}, N_{LIS} and n_{LIS} impregnated with GPL103 and GPL107 in Table SI.III and Table SI.IV, respectively.

Table SI.III –Average droplet shedding radius, $\overline{R}$ (mm), volume, $\overline{V}$ (µl), droplet velocity $\overline{v}$ (µm/s), gravitational force, $\overline{F_g}$ (µN), and pinning force, $\overline{F_{\text{pın-LIS}}}$ (µN), estimated based on Eq. SI.1, and force difference, $\overline{\Delta F_{\text{pın-LIS}}}$ (µN), on MN_{LIS}, mN_{LIS}, N_{LIS} and n_{LIS} impregnated with GPL103.

	$\overline{R}$ (mm)	$\overline{V}$ (µl)	$\overline{v}$ (µm/s)	$\overline{F_g}$ (µN)	$\overline{F_{\text{pın-LIS}}}$ (µN)	$\overline{\Delta F_{\text{pın-LIS}}}$ (µN)
**MN_{LIS}**	0.37 ± 0.11	21.5 ± 22.5	0.21 ± 0.18	2.1 ± 1.8	7.3 ± 2.3	-5.3 ± 2.1
**mN_{LIS}**	0.31 ± 0.07	27.9 ± 20.1	0.12 ± 0.08	1.2 ± 0.8	5.9 ± 1.5	-4.7 ± 1.2
**N_{LIS}**	0.48 ± 0.07	15.8 ± 12.7	0.39 ± 0.15	4.1 ± 1.5	9.0 ± 1.4	-4.9 ± 1.5
**n_{LIS}**	0.54 ± 0.06	14.4 ± 8.2	0.57 ± 0.17	5.6 ± 1.7	10.1 ± 1.1	-4.5 ± 1.4

Table SI.IV –Average droplet shedding radius, $\overline{R}$ (mm), volume, $\overline{V}$ (µl), droplet velocity $\overline{v}$ (µm/s), gravitational force, $\overline{F_g}$ (µN), and pinning force, $\overline{F_{\text{pın-LIS}}}$ (µN), estimated based on Eq. SI.1, and force difference, $\overline{\Delta F_{\text{pın-LIS}}}$ (µN), on MN_{LIS}, mN_{LIS}, N_{LIS} and n_{LIS} impregnated with GPL107.

	$\overline{R}$ (mm)	$\overline{V}$ (µl)	$\overline{v}$ (µm/s)	$\overline{F_g}$ (µN)	$\overline{F_{\text{pın-LIS}}}$ (µN)	$\overline{\Delta F_{\text{pın-LIS}}}$ (µN)
**MN_{LIS}**	0.47 ± 0.15	0.47 ± 0.52	7.6 ± 5.3	4.6 ± 5.1	9.6 ± 3.1	-5.0 ± 4.1
**mN_{LIS}**	0.50 ± 0.1	0.49 ± 0.28	9.5 ± 4.0	4.8 ± 2.7	13.5 ± 2.8	-8.7 ± 2.8
**N_{LIS}**	0.58 ± 0.07	0.71 ± 0.25	7.5 ± 3.5	7.0 ± 2.4	15.4 ± 1.9	-8.4 ± 2.2
**n_{LIS}**	0.50 ± 0.12	0.50 ± 0.35	2.5 ± 2.1	4.9 ± 3.5	10.0 ± 2.5	-5.1 ± 3.0

From Table SI.III and Table SI.IV, pinning forces on hierarchical MN_{LIS} and mN_{LIS}, F_{pin}, are greater than depinning force induce by gravity, F_g, *i.e.*, $\overline{\Delta F_{\text{pın-LIS}}} < 0$. Hence droplet shedding on our LIS is not expected and Equation SI.1 is not able to account for the experimental observations reported in Figure SI.7 and Figure SI.8 (Figure 3a and Figure 3b in the main manuscript). F_{pin} in

Equation SI.1 must therefore account for the effective fraction of the contact line pinned to the uppermost level of the hierarchical roughness, hence $F_{\text{pin-LIS}}$ is now proportional to $\pi\sqrt{\emptyset}D_{\text{b}}$:[2, 16]

$$F_{\text{g}} - F_{\text{pin-LIS}} = \rho V g \sin\alpha - \gamma_{\text{lg}}\pi\sqrt{\phi}D_{\text{b}}(\cos\theta_{\text{r}} - \cos\theta_{\text{a}}) \qquad \text{Eq. SI.2}$$

where $D_b = D\sin\theta_a$, while f is the effective pinned fraction. The effective pinned fraction on N_{LIS} and n_{LIS} is obtained from Table SI.I as $\phi_{\text{nano}} = f$. On the other hand, in the case of MN_{LIS} and mN_{LIS}, ϕ equals f times the solid fraction of the microstructures Ω as $\phi_{\text{nano}} = f\Omega$. Ω is extracted from Figure SI.2 and Figure SI.3 and equals 0.305 and 0.196 on MN_{LIS} and mN_{LIS}, respectively. Table SI.V (Table II in the main manuscript) and Table SI.VI (Table III in the main manuscript) includes a revisited $F_{\text{pin-LIS}}$ for the averaged droplet shedding radius from experimental observations reported in Figure SI.7 (Figure 2a in the main manuscript) and Figure SI.8 (Figure 2b in the main manuscript) for GPL103 and GPL107, respectively:

Table SI.V –Average droplet shedding radius, $\bar{R}$ (mm), volume, $\bar{V}$ (µl), droplet velocity $\bar{v}$ (µm/s), gravitational force, $\overline{F_g}$ (µN), and pinning force, $\overline{F_{\text{pin-LIS}}}$ (µN), estimated based on Eq. SI.2, on MN_{LIS}, mN_{LIS}, N_{LIS} and n_{LIS} impregnated with GPL103.

	$\bar{R}$ (mm)	$\bar{V}$ (µl)	$\bar{v}$ (µm/s)	$\overline{F_g}$ (µN)	$\overline{F_{\text{pin-LIS}}}$ (µN)	$\overline{\Delta F_{\text{pin-LIS}}}$ (µN)
**MN_{LIS}**	0.37 ± 0.11	0.21 ± 0.18	21.5 ± 22.5	2.1 ± 1.8	1.3 ± 0.4	0.8 ± 1.1
**mN_{LIS}**	0.31 ± 0.07	0.12 ± 0.08	27.9 ± 20.1	1.2 ± 0.8	0.9 ± 0.2	0.3 ± 0.5
**N_{LIS}**	0.48 ± 0.07	0.39 ± 0.15	15.8 ± 12.7	4.1 ± 1.5	3.0 ± 0.5	1.1 ± 1.0
**n_{LIS}**	0.54 ± 0.06	0.57 ± 0.17	14.4 ± 8.2	5.6 ± 1.7	3.2 ± 0.4	2.4 ± 1.1

Table SI.VI –Average droplet shedding radius, $\bar{R}$ (mm), volume, $\bar{V}$ (µl), droplet velocity $\bar{v}$ (µm/s), gravitational force, $\overline{F_g}$ (µN), and pinning force, $\overline{F_{\text{pin-LIS}}}$ (µN), estimated based on Eq. SI.2, on MN_{LIS}, mN_{LIS}, N_{LIS} and n_{LIS} impregnated with GPL107.

	$\bar{R}$ (mm)	$\bar{V}$ (µl)	$\bar{v}$ (µm/s)	$\overline{F_g}$ (µN)	$\overline{F_{\text{pin-LIS}}}$ (µN)	$\overline{\Delta F_{\text{pin-LIS}}}$ (µN)
**MN_{LIS}**	0.47 ± 0.15	0.47 ± 0.52	7.6 ± 5.3	4.6 ± 5.1	1.8 ± 0.6	1.3 ± 0.4
**mN_{LIS}**	0.50 ± 0.1	0.49 ± 0.28	9.5 ± 4.0	4.8 ± 2.7	2.0 ± 0.4	0.9 ± 0.2
**N_{LIS}**	0.58 ± 0.07	0.71 ± 0.25	7.5 ± 3.5	7.0 ± 2.4	5.1 ± 0.6	3.0 ± 0.5
**n_{LIS}**	0.50 ± 0.12	0.50 ± 0.35	2.5 ± 2.1	4.9 ± 3.5	3.2 ± 0.9	3.2 ± 0.4

Results from Table SI.V (Table II in the main manuscript) and Table SI.VI (Table III in the main manuscript) confirm that droplet adhesion on LISs during dynamic condensation can be eventually reduced by the presence of micro-structures that effectively decrease the effective fraction of pinned contact line. And for the drops observed, the force of gravity now overcomes that of pinning inducing the observed shedding of droplets with diameters in the submillimetre range.

SI.9 – DROPLET GROWTH RATES

Droplet growth follows a typical power law relation as: $\langle D \rangle \propto At^{\mu}$, where $\langle D \rangle$ is the average diameter, A is a constant, t is time and μ is the power law exponent ranging from 0 to 1.[15, 17, 18] For small droplets condensing and growing with diameters **between 2 and 30 µm** the constant A and the power law exponent μ are summarized in Table SI.VII, for MN_{LIS} and N_{LIS} impregnated with GPL103 and GPL107:

Table SI.VII –Average and standard deviation of droplet growth $\langle D \rangle$ as At^{μ} between 2 and 30 µm, on MN_{LIS}, N_{LIS} impregnated with GPL103 and GPL107 at different intervals of time, *i.e.*, *t* = 1 – 5 mins, *t* = 5 – 15 mins, and *t* = 15 – 30 mins:

$\langle D \rangle \propto At^{\mu}$	*t* = 1 – 5 mins	*t* = 5 – 15 mins	*t* = 15 – 30 mins
**MN_{LIS_GPL103}**	$(2.2 \pm 0.3)\ t^{(0.54 \pm 0.01)}$	$(2.4 \pm 0.3)\ t^{(0.53 \pm 0.01)}$	$(2.3 \pm 0.3)\ t^{(0.53 \pm 0.05)}$
**N_{LIS_GPL103}**	$(2.6 \pm 0.6)\ t^{(0.55 \pm 0.01)}$	$(2.6 \pm 0.4)\ t^{(0.51 \pm 0.06)}$	$(2.4 \pm 0.2)\ t^{(0.49 \pm 0.02)}$
**MN_{LIS_GPL107}**	$(2.4 \pm 0.2)\ t^{(0.50 \pm 0.02)}$	$(2.4 \pm 0.2)\ t^{(0.47 \pm 0.03)}$	$(2.5 \pm 0.1)\ t^{(0.45 \pm 0.01)}$
**N_{LIS_GPL107}**	$(2.9 \pm 0.1)\ t^{(0.51 \pm 0.02)}$	$(3.5 \pm 0.1)\ t^{(0.48 \pm 0.05)}$	$(3.4 \pm 0.1)\ t^{(0.46 \pm 0.02)}$

From Table SI.VII, the average droplet growth exponent is ca. 0.501 with ± 0.032 as standard deviation and the coefficient A equals 2.6 ± 0.5, independently of the surface structure, type of oil or interval of time studied.

When looking into GPL103 at different growth intervals named direct condensation between 2 and 30 µm, condensation-coalescence between 30 and 200 µm, condensation-coalescence between 200 and the average shedding diameter and for sizes greater than the shedding size; the relationship $\langle D \rangle \propto At^{\mu}$ can be extracted and plotted in Table SI. VIII:

Table SI.VIII –Average and standard deviation of droplet growth $\langle D \rangle$ as At^{μ} on MN_{LIS}, mN_{LIS}, N_{LIS} and n_{LIS}, impregnated with GPL103 for the different growth regimes reported namely: direct condensation, condensation-coalescence and condensation-coalescence-shedding. Note that we also divide condensation-coalescence regime into microscopic for droplet sizes between 30 µm and 200 µm in diameter and macroscopic for droplet sizes between 200 µm and the droplet shedding size.

$\langle D \rangle \propto At^{\mu}$	Direct condensation	Condensation-coalescence		Condensation-coalescence-shedding
		30 µm - 200 µm	*200 µm - shedding*	
**MN_{LIS_GPL103}**	$(2.3 \pm 0.1)\ t^{(0.53 \pm 0.01)}$	$(0.25 \pm 0.04)\ t^{(1.02 \pm 0.04)}$	$(0.99 \pm 0.28)\ t^{(0.81 \pm 0.03)}$	$(1.0 \pm 2.0)\cdot 10^{-23}\ t^{(13.5 \pm 4.9)}$
**mN_{LIS_GPL103}**	$(2.5 \pm 0.1)\ t^{(0.53 \pm 0.01)}$	$(0.15 \pm 0.14)\ t^{(1.26 \pm 0.08)}$	$(0.35 \pm 0.15)\ t^{(1.00 \pm 0.07)}$	$(1.8 \pm 3.0)\cdot 10^{-24}\ t^{(16.0 \pm 4.9)}$
**N_{LIS_GPL103}**	$(2.5 \pm 0.1)\ t^{(0.52 \pm 0.03)}$	$(0.16 \pm 0.07)\ t^{(1.13 \pm 0.07)}$	$(0.36 \pm 0.10)\ t^{(0.96 \pm 0.02)}$	$(1.8 \pm 3.6)\cdot 10^{-14}\ t^{(17.2 \pm 15.1)}$
**n_{LIS_GPL103}**	$(2.5 \pm 0.1)\ t^{(0.52 \pm 0.03)}$	$(0.28 \pm 0.09)\ t^{(1.04 \pm 0.10)}$	$(0.98 \pm 0.55)\ t^{(0.90 \pm 0.11)}$	$(1.7 \pm 3.7)\cdot 10^{-33}\ t^{(14.4 \pm 3.0)}$

SI.10 –HEAT TRANSFER CONSIDERATIONS

After computing the different droplet growth rates for the different regimes highlighted above and extending the results up to 12,500 seconds, which is equivalent to 3 to 6 droplet cycles depending on the surface studied, the cumulative heat transfer function of time can be obtained and presented in Figure SI.9:

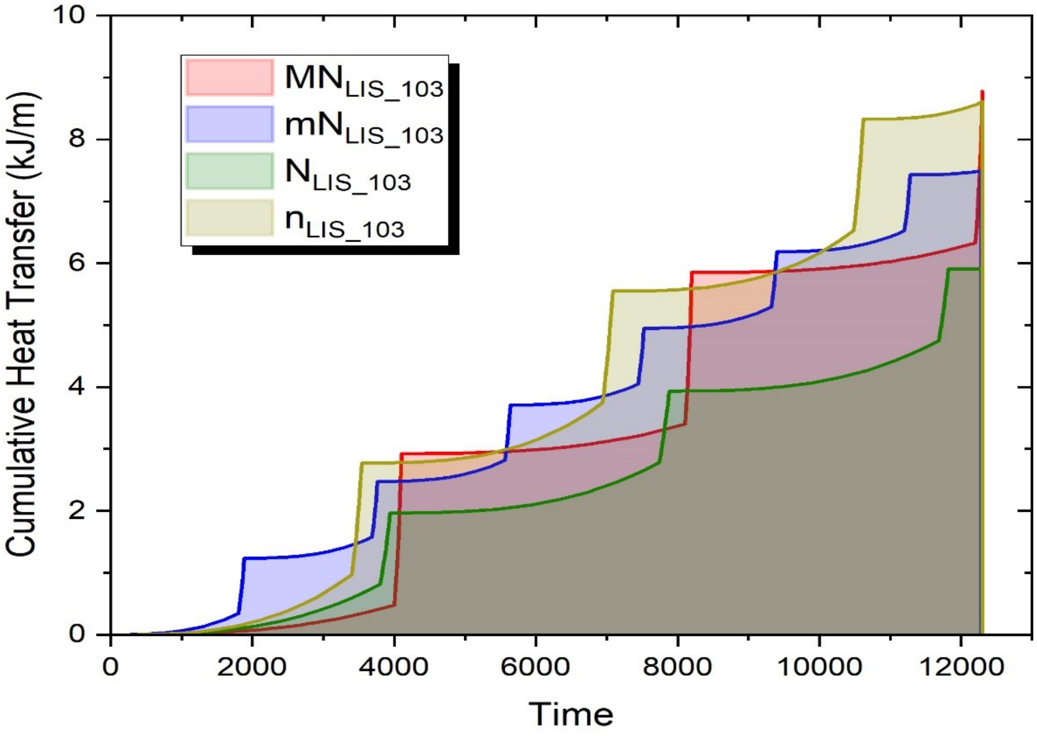

Figure SI.9 – Cumulative heat transfer per unit length (kJ/m) versus time *t* (seconds) for MN_{LIS_103}, mN_{LIS_103}, N_{LIS_103} and n_{LIS_103}.

Despites the 100% lower heat transfer performance reported when comparing MN_{LIS} and mN_{LIS} to N_{LIS} and n_{LIS}[1], the better shedding performance of MN_{LIS} and mN_{LIS} as a consequence of the presence of micro-structures is able to achieve cumulative heat transfer per unit length values of the same order of magnitude as reported in Figure SI.9. MN_{LIS} and mN_{LIS} better shedding performance is able to induce a greater droplet growth in the condensation-coalescence-shedding regime and hence cumulative heat transfer coefficients of the same order of magnitude as in the case of N_{LIS} and n_{LIS}. Figure SI.9 includes the cumulative heat transfer per unit length (kJ/m) versus time (s) for all 4 LISs.

REFERENCES

1. Maeda, Y., et al., *Condensate droplet size distribution and heat transfer on hierarchical slippery lubricant infused porous surfaces.* Applied Thermal Engineering, 2020. **176**: p. 115386.
2. Smith, J.D., et al., *Droplet mobility on lubricant-impregnated surfaces.* Soft Matter, 2013. **9**(6): p. 1772-1780.
3. Bico, J., U. Thiele, and D. Quéré, *Wetting of textured surfaces.* Colloids and Surfaces A: Physicochemical and Engineering Aspects, 2002. **206**(1): p. 41-46.
4. Guan, J.H., et al., *Evaporation of Sessile Droplets on Slippery Liquid-Infused Porous Surfaces (SLIPS).* Langmuir, 2015. **31**(43): p. 11781-11789.
5. Zhang, P., et al., *Enhanced Coalescence-Induced Droplet-Jumping on Nanostructured Superhydrophobic Surfaces in the Absence of Microstructures.* ACS Applied Materials & Interfaces, 2017. **9**(40): p. 35391-35403.
6. Quéré, D., *Wetting and Roughness.* Annual Review of Materials Research, 2008. **38**(1): p. 71-99.
7. ImageJ, I.b.W.R., National Institutes of Health, USA, last checked: April 2016, URL http://imagej.nih.gov/ij.
8. Stalder, A.F., et al., *A snake-based approach to accurate determination of both contact points and contact angles.* Colloids and Surfaces A: Physicochemical and Engineering Aspects, 2006. **286**(1): p. 92-103.
9. Daerr, A. and A. Mogne, *Pendent_Drop: An ImageJ Plugin to Measure the Surface Tension from an Image of a Pendent Drop.* Open Research Software, 2016. **4**(1): p. e3.
10. Dai, X., et al., *Slippery Wenzel State.* ACS Nano, 2015. **9**(9): p. 9260-9267.
11. Carre, A. and M.E.R. Shanahan, *Drop Motion on an Inclined Plane and Evaluation of Hydrophobia Treatments to Glass.* The Journal of Adhesion, 1995. **49**(3-4): p. 177-185.
12. Kim, H.-Y., H.J. Lee, and B.H. Kang, *Sliding of Liquid Drops Down an Inclined Solid Surface.* Journal of Colloid and Interface Science, 2002. **247**(2): p. 372-380.
13. Olsen, D.A., P.A. Joyner, and M.D. Olson, *The Sliding of Liquid Drops on Solid Surfaces.* The Journal of Physical Chemistry, 1962. **66**(5): p. 883-886.
14. Furmidge, C.G.L., *Studies at phase interfaces. I. The sliding of liquid drops on solid surfaces and a theory for spray retention.* Journal of Colloid Science, 1962. **17**(4): p. 309-324.
15. Sharma, V., et al., *Gladiolus dalenii Based Bioinspired Structured Surface via Soft Lithography and Its Application in Water Vapor Condensation and Fog Harvesting.* ACS Sustainable Chemistry & Engineering, 2018. **6**(5): p. 6981-6993.
16. Paxson, A.T. and K.K. Varanasi, *Self-similarity of contact line depinning from textured surfaces.* Nature Communications, 2013. **4**: p. 1492.
17. Beysens, D. and C.M. Knobler, *Growth of Breath Figures.* Physical Review Letters, 1986. **57**(12): p. 1433-1436.
18. Chavan, S., et al., *Heat Transfer through a Condensate Droplet on Hydrophobic and Nanostructured Superhydrophobic Surfaces.* Langmuir, 2016. **32**(31): p. 7774-7787.
